# Added Value of Reanalysis of Whole Exome- and Whole Genome Sequencing Data From Patients Suspected of Primary Immune Deficiency Using an Extended Gene Panel and Structural Variation Calling

**DOI:** 10.3389/fimmu.2022.906328

**Published:** 2022-06-30

**Authors:** Sara Bohnstedt Mørup, Lusine Nazaryan-Petersen, Migle Gabrielaite, Joanne Reekie, Hanne V. Marquart, Hans Jakob Hartling, Rasmus L. Marvig, Terese L. Katzenstein, Tania N. Masmas, Jens Lundgren, Daniel D. Murray, Marie Helleberg, Line Borgwardt

**Affiliations:** ^1^ Centre of Excellence for Health, Immunity, and Infections, Rigshospitalet, Copenhagen University Hospital, Copenhagen, Denmark; ^2^ Center for Genomic Medicine, Rigshospitalet, Copenhagen University Hospital, Copenhagen, Denmark; ^3^ Department of Clinical Immunology, Rigshospitalet, Copenhagen University Hospital, Copenhagen, Denmark; ^4^ Department of Infectious Diseases, Rigshospitalet, Copenhagen University Hospital, Copenhagen, Denmark; ^5^ The Child and Adolescent Department, Rigshospitalet, Copenhagen University Hospital, Copenhagen, Denmark

**Keywords:** structural variation analysis, primary immune deficiencies (PID), single nucleotide variant analysis, whole genome sequencing (WGS), reanalysis approach, whole exome sequencing, small INDELs, gene panel analysis

## Abstract

**Background:**

Knowledge of the genetic variation underlying Primary Immune Deficiency (PID) is increasing. Reanalysis of genome-wide sequencing data from undiagnosed patients with suspected PID may improve the diagnostic rate.

**Methods:**

We included patients monitored at the Department of Infectious Diseases or the Child and Adolescent Department, Rigshospitalet, Denmark, for a suspected PID, who had been analysed previously using a targeted PID gene panel (457 PID-related genes) on whole exome- (WES) or whole genome sequencing (WGS) data. A literature review was performed to extend the PID gene panel used for reanalysis of single nucleotide variation (SNV) and small indels. Structural variant (SV) calling was added on WGS data.

**Results:**

Genetic data from 94 patients (86 adults) including 36 WES and 58 WGS was reanalysed a median of 23 months after the initial analysis. The extended gene panel included 208 additional PID-related genes. Genetic reanalysis led to a small increase in the proportion of patients with new suspicious PID related variants of uncertain significance (VUS). The proportion of patients with a causal genetic diagnosis was constant. In total, five patients (5%, including three WES and two WGS) had a new suspicious PID VUS identified due to reanalysis. Among these, two patients had a variant added due to the expansion of the PID gene panel, and three patients had a variant reclassified to a VUS in a gene included in the initial PID gene panel. The total proportion of patients with PID related VUS, likely pathogenic, and pathogenic variants increased from 43 (46%) to 47 (50%), as one patient had a VUS detected in both initial- and reanalysis. In addition, we detected new suspicious SNVs and SVs of uncertain significance in PID candidate genes with unknown inheritance and/or as heterozygous variants in genes with autosomal recessive inheritance in 8 patients.

**Conclusion:**

These data indicate a possible diagnostic gain of reassessing WES/WGS data from patients with suspected PID. Reasons for the possible gain included improved knowledge of genotype-phenotype correlation, expanding the gene panel, and adding SV analyses. Future studies of genotype-phenotype correlations may provide additional knowledge on the impact of the new suspicious VUSs.

## Introduction

Primary immune deficiency (PID) is defined as immune dysfunction due to a genetic cause, which causes either inhibition and/or gain of function of components within the innate and/or adaptive immune system (1). The prevalence of patients with PID is estimated to be as high as one out of every thousand individuals (2). Patients with PID have impacted quality of life and increased mortality primarily due to infectious complications (3). Currently the total number of different monogenetic PID disorders extends to over 400 and is continuously increasing (4). Nevertheless, targeted analysis of PID genes on whole exome- or whole genome sequencing (WES/WGS) data detects monogenetic pathogenic variants in less than 50% of patients suspected of PID (5, 6). Due to the continuously increasing knowledge of genes and variants related to PID, a reanalysis of prior WES/WGS data with an extended PID gene panel in undiagnosed patients may improve the diagnostic performance. The potential benefit of performing reanalysis needs to be established, so that institutes can properly weigh up cost benefit.

There are wide ranging benefits of providing a genetic diagnosis for the individual patients. The confirmation or re-classification of a clinical diagnosis may provide the potential for treatment adjustments. In addition, a clarification of the inheritance pattern is important to provide genetic counselling for relatives at risk, as well as the opportunity for prenatal or preimplantation genetic tests in relevant cases. Furthermore, the gain in knowledge of the underlying causes of PID diseases can pave the way for the development of novel biomarkers in risk stratification and treatments. Therefore, reanalysis of WES/WGS data is important to consider in previously undiagnosed cases. In other disease groups, conducting a semiautomated reanalysis of WES/WGS data have been shown to increase the rate of genetic diagnoses (7–10), primarily due to the analysis of newly added genes (11–13).

In this study, we aimed to assess the increase in the diagnostic rate after reanalysis using an extended gene panel on WES/WGS data in a cohort consisting of patients over 16 years of age and suspected of PID. The reanalyses included a repeated analysis for single nucleotide variants (SNVs) and small indels in 94 patients and adding analysis for structural variants (SVs) in 56 patients analysed with WGS. We investigate the proportion of new genetic variants identified after the reanalysis due to either 1) improved knowledge of phenotype-genotype correlation leading to a variant re-classification 2) expansion of the PID gene panel or 3) addition of SV analysis. To our knowledge, this is the first study conducting a WES/WGS data reanalysis in a PID population.

## Materials and Methods

### Patient Population

**Figure 1:**
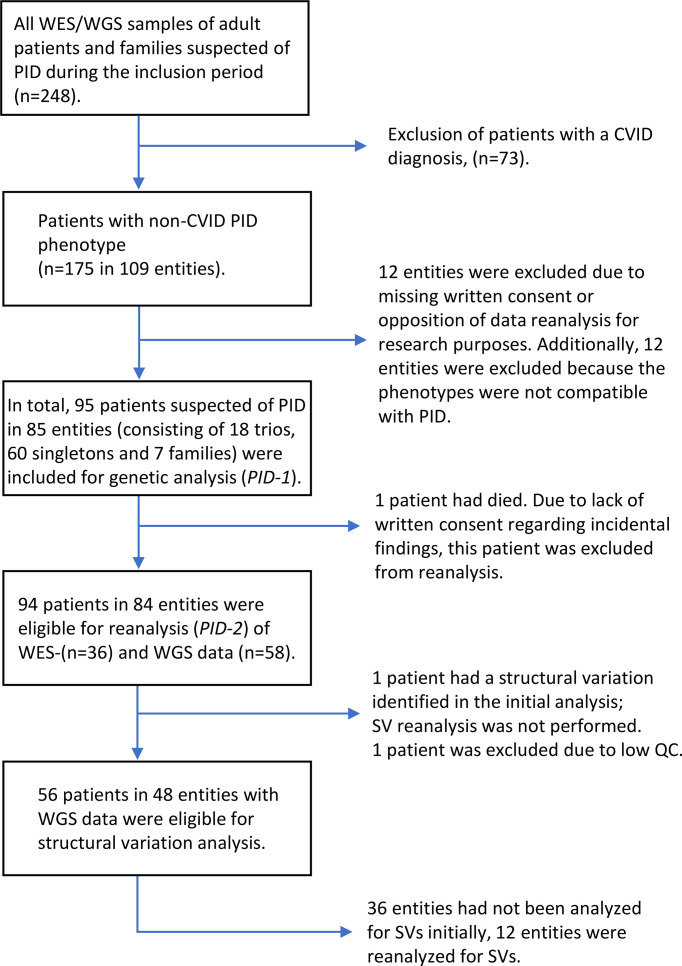
Flowchart of patient inclusion.

Patients monitored at the Department of Infectious Diseases, Rigshospitalet, Copenhagen, Denmark, for a suspected PID between January 2016 and September 2020 were offered WES/WGS analysed with a targeted PID gene panel (*PID-1*), after clinical evaluation and most often also following immunological analyses. Patients who accepted the offer and gave informed consent were included in the study (**Figure 1**). The study was approved by the national ethics committee. Patients fulfilling the diagnostic criteria for Common Variable Immunodeficiency (CVID) (14, 15) were subsequently excluded from this analysis, as a separate prospective study focused on CVID is ongoing. However, one child, who fulfilled the diagnostic criteria for CVID, was included as the child was part of a family including a parent and sibling with antibody deficiency. The excluded patients with CVID constituted approximately half of the patients suspected of PID and referred for genetic analysis at our institute.

Phenotype data were collected from patients records and assigned the Human Phenotype Ontology (HPO) terms (16). Patients enrolled between January 2016 and January 2018 were analysed with WES (n=37) and patients enrolled between January 2018 and September 2020 were analysed with WGS (n=58). One of the patients analysed with WES, who had a causal pathogenic variant and had died, was excluded from reanalysis. In total 94 patients had WES/WGS data reanalysed for SNVs and small indels. Two of the patients with WGS data were excluded from SV analysis, one of these had poor quality control of WGS data and one had a causal pathogenic SV reported in the initial analysis. In total, WGS data from 56 patients were analysed for SVs. The genetic analysis consisted of three different types of entities: 1) 18 Trio analysis (the analysis of a patient and the patient’s parents), 2) 60 Singletons (one patient analysis) and 3) Seven Families (one patient and one or more relatives also presenting symptoms compatible with a PID).

### Extended PID Gene Panel

The initial gene panel, PID-1, was developed in 2015 and contained 457 genes. PID-1 genes were primarily from the 2016 European Society for Immunodeficiencies online registry of diseases genes (17) and Practice parameter for the diagnosis and management of primary immunodeficiency (18). References included in the development of PID-1 are listed in **Supplementary**. Based on a literature search in October 2020, 208 genes were added resulting in a total of 665 genes in PID-2. The majority of genes were added from Genomics England Panel App (19) (**Supplementary**). PID-1 and PID-2 included both known disease-associated PID genes and candidate genes for which the association with PID needs further study. PID-2 was implemented for clinical use in our laboratory after 1 November 2020 (**Supplementary Table 1**).

### Whole Exome Sequencing

Genomic DNA was extracted from whole blood. 500 ng of DNA was fragmented to an average size of 300 base pairs using Covaris S2 (Woburn, MA, USA). Adaptor ligation was performed using the KAPA HTP Library Preparation Kit (Roche Diagnostics, Basel, Switzerland) on a Sciclone G3 robot (Perkin Elmer, Waltham, MA, USA). Sequence capture targeting the exome was done with the SureSelectXT Clinical Research Exome kit (Agilent) and followed sequencing on HiSeq2500 or NextSeq500 (Illumina, San Diego, CA, USA). For WES the sequencing protocol aimed to achieve at least 95% of positions within the capture covered by minimum 10x and average coverage of 50x.

### Whole Genome Sequencing

Genomic DNA was isolated from whole blood samples using the liquid handling automated station (Tecan Freedom EVO HSM 2.0 Workstation) according to the manufacturer’s instructions (Promega Corporation, USA, ReliaPrep Large Volume HT gDNA Isolation System). Genomic DNA was subjected to WGS using Nextera DNA Flex library preparation kit and sequenced on a Novaseq 6000 (Illumina, San Diego, CA, USA). For WGS the aim was for at least 98% of mappable positions to be covered by minimum 10x and average coverage of 30x, which was achieved for nearly all samples. Four samples were borderline with a sequence coverage range of 95- 97% (*28S, 36S, 57S, 83T*). One patient (*15FC1*) was excluded from SV analysis due to low coverage.

### Variant Calling

Single nucleotide variants and small insertions and deletions were called with GATK Haplotype Caller version 4.1.3.0 in accordance with the GATK best practice guidelines developed by the Broad Institute (20). Structural variants were called by Manta version 1.5.0 (21), Lumpy version 0.3.0 (22), and CNVnator version 0.3.3 (23). Thereafter, sequences called with at least two of three tools were manually inspected.

### Variant Exploration and Classification

For both WES and WGS datasets variant filtering was performed using the Ingenuity Variant Analysis or QCI software (Qiagen Bioinformatics, Redwood City, CA). Integrative Genomics Viewer (IGV) Genome Browser (24) was used to manually verify findings in the aligned raw sequencing data. The variant calling was genotype driven as the PID gene panels were used for all patients. In a minor proportion of patients all exome or genome data was analysed, or disease specific search words were applied in Ingenuity. In the trios and families, the information of family members was included in the filtering and interpretation of variants. In SNV and small indels analysis only variants with a minor allele frequency of less than 2% in GnomAD (25) data and variants in coding regions (+/− 20 base pairs) were selected for further analysis unless an established Pathogenic common variant, reported pathogenic in The Human Gene Mutation Database (26) or ClinVar (27) or predicted a splice effect by MaxEntScan (28). In the analysis of SNVs and small indels we reanalysed WES/WGS data from all patients with *PID-2* and used the phenotypic information to evaluate the relevance of variants. For SVs, we used the same +/- 20 bp for intron boundaries and +/- 10 kb for the 1^st^ exon of the genes for promoter/regulatory regions. By WGS-based SV-analysis we could pick up as small as 30bp SVs. SV analysis on WGS data was performed genome wide as an explorative analysis. Only SVs affecting genes included in *PID-2* and/or genes related to key phenotypic findings were reported. As a standard laboratory procedure, the variants of interest were not verified by Sanger sequencing unless they did not fulfil the required quality measures (coverage, variant allele frequency etc.). The variants were classified in accordance with the American College of Medical Genetics and Genomics and the Association for Molecular Pathology (ACMG/AMP) guidelines (29, 30). For each patient, data was analysed and variants were classified by two molecular biologists and/or clinical geneticists.

### Immunological Analysis

The standard immunological analysis included flow cytometry-based leukocyte count and subtyping of maturation- and activation stages, somatic hypermutation (measuring in kappa light chain transcripts), T-lymphocyte proliferation (for patients between the ages of 0 and 10 or for adults suspected of a T-cell defect) and complement activation screen. Further specific immunological analyses were performed based on the patient's clinical presentation and results of the basic immunological analysis after conference with clinical immunologists (31).

## Results

### Patient Population

In total, 95 individuals suspected of having PID in 85 entities consisting of 18 trios, 60 singletons and 7 families were included in the study. Patients were categorized and sub-grouped according to ESID (2019) clinical criteria (17) based on the presentation at the time of the initial genetic analysis (*PID-1*). The patients´ clinical and immunological phenotypes were assessed again prior to the reanalysis in order to include any additional information obtained between the initial- and reanalysis. Baseline characteristics are presented in **Table 1** and detailed phenotype data is in **Supplementary Table 2**. The major clinical subgroups in our PID cohort were unclassified antibody deficiencies and unclassified autoinflammatory diseases. The median time between the initial- and the reanalysis of WES/WGS data was 23 months (range 1-57 months).

**Table 1:** Baseline Characteristics.

Total number of patients, (n)	95
Adults, ≥18 years (n), Children (n)	87, 8
Age at inclusion (years), median (range)	37 (16-75)
Symptom onset at age 0-10 years (n (%))	44 (46)
Symptom onset at age 10-20 years (n (%))	12 (13)
Symptom onset at age 20-40 years (n (%))	18 (19)
Symptom onset at age > 40 years (n (%))	20 (21)
Symptom onset at unknown age	1 (1)
Female (n (%))	65 (68)
Time between the initial- and the reanalysis of WES/WGS data (months), median (range)	23 (1-57)
PID related Clinical Characteristics^*^	
Recurrent/opportunistic infections (n (%))	78 (81)
Autoimmunity (n (%))	15 (16)
Malignancy (n (%))	5 (5)

^*^Patients can have more than one PID related clinical characteristic registered.

PID, Primary immune deficiency; WES, Whole Exome Sequencing; WGS, Whole Genome Sequencing.

### Results of Genetic Analysis

The results of the initial genetic analyses (*PID-1*) and reanalyses (*PID-2*) are presented in **Supplementary Table 3** and **Table 2**, respectively. The number of patients with genetic findings identified before and after reanalysis are summarized in **Table 3** and described further below. In the initial analysis, a causal PID diagnosis was detected in eight patients, including likely pathogenic- (LP) and pathogenic (P) variants (**Supplementary Table 3**). One of these eight patients was excluded from reanalysis. No new PID diagnoses were detected upon reanalysis. Seven of the patients with a genetic diagnosis had a SNV or small indel (*11S, 25S, 44T, 45S, 46S, 48T, 74T*) and one patient had a SV (*63S*). One patient (*52S*) had a variant of uncertain significance (VUS) in *NLRP3*, which was compatible with the phenotype and segregated in the affected family members. Due to reduced penetrance of the phenotype this variant could not be classified as LP or P. Therefore, this patient was not counted as having a genetic diagnosis. Additionally, in the initial analysis eight patients (*5S, 37S, 53S, 54S, 62FP, 62FC, 77S, 81S*) had suspicious PID variants detected (LP/P), which were not explanatory for the PID phenotype (including heterozygous variants in PID genes with AR inheritance). Further, 28 patients had suspicious PID VUS(s) detected. In the reanalysis, five (5%) patients had new suspicious PID related VUS(s) identified. Four out of the five patients did not have any variant identified in the initial analysis. Due to the addition of VUSs, the reanalysis increased the total number of patients in the cohort with PID related VUS(s), LP, and P variant(s) from 43 (46%) to 47 (50%).

**Table 2:** Variants detected after reanalysis with PID-2 and analysis for structural variation.

ID^*^	WES/WGS	Genetic result of SNV or SV analysis (gene, cDNA, Protein, RefSeq)	ACMG classification^‡^	SNV/SV predicted coding effect	Zygosity	Inheritance	Allele frequency for NFE in GnomAD (%), and for SV DGV (%), (All)	CADD	Previous report of variant in HGMD (PMID)	Gene function or pathway involvement (PMID)	Comment	Category of reanalysis responsible for the detection of the new finding^§^
Unclassified Antibody Deficiency												
7S	WGS	*GSN* (c.487G>A, p.(Asp163Asn), NM_001353054.1)^†^	3	Missense	Het	Unknown	0.001	26.5	_	Actin-binding protein (PMID: 30698126)	*GSN* gene function potentially related to multiple sclerosis (PMID: 21040581)	_
9S	WGS	*BANK1* (c.723del, p.(Met241Ilefs*35), NM_001127507.2)	3	Frameshift	Het	Unknown	0	N/A	_	B-cell receptor-, CD40-related-, TLR7- and TLR9 signaling (PMID: 34066164)	_	4
10FP	WGS	chr9:g.73297_381074dup Tandem duplication on 9p24.3 (~300kb) including exons 1-21 of *DOCK8* (NM_203447.4)	3	_	Het	AR	DGV: 0.14	_	_	TCR (PMID: 27599296), TLR9 (PMID: 28882618)	The duplication is not present in the son (10FC).	4
Selective IgM Deficiency												
16S	WES	*NFKBID* (c.359C>T, p.(Ser120Leu), NM_139239.3)	3	Missense	Het	Unknown	<0.001	24.5	_	NF-κB (PMID: 23578005)	_	4
IgG-Subclass Deficiency												
19S	WGS	*FLG* (c.10255C>T, p.(Arg3419*), NM_002016.2)	3	Nonsense	Het	AD/AR	0.05	39	PMID: 31365035	Protein in the skin barrier (PMID: 31509236)	_	1
T-cell Deficiency and Combined Immunodeficiency												
27S	WGS	*LAG3* (c.287_407 del, p.(Pro96Argfs*36), NM_002286.6) chr12:g.6882943_6883063del A 135bp deletion in exon 3 of *LAG3*	3	Frameshift	Het	Unknown	GnomAD-SV: 0, DGV 0	_	_	T-cell receptor signaling pathway (PMID: 31848144)	_	4
29S	WES	*OAS1* (c.163G>A, p.(Val55Met), NM_002534.3)	3	Missense	Het	AD	0.006	23	_	Negative regulator of the expression of chemokines and interferon responsive genes in human macrophages (PMID: 30078389). RNA cleavage pathway (PMID: 30822544)	_	2
31S	WGS	*TNFAIP3* (c.2140C>A, p.(Pro714Thr), NM_006290.4)	3	Missense	Het	AD	0	17.19	_	NF-κB (PMID: 32334614)	_	2
32S	WGS	chr9:g.72599_452686dup Tandem duplication on 9p24.3 (~400kb) including exons 1-44 of *DOCK8* (NM_203447.4)	3	_	Het	AR	DGV: 0.11	_	_	TCR (PMID: 27599296), TLR9 (PMID: 28882618)	_	4
Pathogen-Specific Immunodeficiency												
33S	WGS	*MAVS* (c.1186C>T, p.(Arg396Trp), NM_001206491.2)	3	Missense	Het	Unknown	0.007	23.9	_	Adaptor protein in RIG-I pathway (PMID: 25636800)	Unknown whether SV and SNV are in cis or trans.	4
33S	WGS	chr20:g.3836764_3840471del, ~4kb deletion including exon 3 of *MAVS* (c.118_292del, p.(Asp40Glyfs*93), NM_001206491.2)	3	Frameshift	Het	Unknown	GnomAD-SV: 0, DGV 0	_	_	Adaptor protein in RIG-I pathway (PMID: 25636800)	The deletion is mediated by *AluY* elements; therefore, it is not possible to identify the exact genomic coordinates.	_
39FA1	WGS	*FPR2* (c.220T>C, p.(Phe74Leu), NM_001462.3)	3	Missense	Het	Unknown	0.0046	23	_	Annexin A1/FPR2 (PMID: 31908042)	First degree relative with resembling phenotype does not carry the missense variant.	4
39FA1	WGS	chr19:g.52271806_52617740dup Tandem duplication on 19q13.41 (~300kb) including exon 2 of *FPR2* (NM_001462.3) and the entire *FPR3* gene (NM_002030.5)	3	_	Het	Unknown	GnomAD-SV: 0.037, DGV: 0,22	_	_	Annexin A1/FPR2 (PMID: 31908042)	Unknown whether SV and SNV are in cis or trans.	_
Autoinflammatory Disorders and Periodic Fever Syndromes												
49S	WES	*NFKB1* (c.689G>T, p.(Arg230Leu), NM_003998.4)	3	Missense	Het	AD	≤ 0.001	22.7	_	NF-κB (PMID: 26663363)	_	1
59S	WES	*SERPING1* (c.5C>T, p.(Ala2Val), NM_000062.3)	3	Missense	Het	AD/AR	0.11	23.6	PMID: 32896191	C1-inhibitor. Major control of kallikrein-kinin system (PMID: 31517426)	_	1
Patients not fulfilling ESID (2019) Criteria												
75S	WGS	chrX:g.68686839_69193319del ~500 deletion on Xq13.1 including exons 1-2 of *EDA* (NM_001399.5)^†^	5	_	Hem	XLR	GnomAD-SV: 0, DGV 0	_	PMID: 8696334	EDA/EDAR/NF-κB and TNFα pathways (PMID: 29855039, PMID: 12351572)	_	_
75S	WGS	*IKBKE* (c.446+5G>A, NM_001193321.2)	3	Splice effect	Het	Unknown	0.0022	16.63	*Clinvar:* likely benign	Oncogenic protein, activates NF-κB pathway (PMID: 31733040)	_	4

All genomic coordinates are given according to hg19 reference genome. ^*^S, Singleton; T, Trio; F, Family; P, Parent; A, Adult in family without included children. ^†^Incidental findings. ^‡^ACMG classification: 3 = variant of uncertain significance (VUS), 4 = likely pathogenic (LP), and 5 = pathogenic (P). ^§^Categories of the reanalysis responsible for detection of the new finding: 1 = improved knowledge of genotype-phenotype, 2 = expansion of PID gene panel, 3 = SV analysis., or 4 = VUS(s) in candidate genes or heterozygous VUS(s) in genes with AR inheritance.

AD, Autosomal dominant; AR, Autosomal recessive; CADD, Combined Annotation Dependent Depletion; Hem, Hemizygous; Het, Heterozygous; HGMD, Human Gene Mutation Database; Ho, Homozygous; NFE, GnomAD Non-Finish European population (All patients with genetic findings are NFE); PMID, PID, Primary immune deficiency; PubMed Identifier; SNV, Single nucleotide variant or small indel; SV, Structural variant; WES, Whole Exome Sequencing; WGS, Whole Genome Sequencing; XLR, X-linked Recessive.

**Table 3:** Number of variants of uncertain significance (VUS), likely pathogenic- (LP) and pathogenic variants (P) before and after reanalysis (n = 94).

	Total patients	Patients with variants identified in the initial analysis (*PID-1)*	Patients with new variants identified in the reanalysis (*PID-1 & PID-2*)
	n (%)	n (%)	n (%)
Genetic diagnosis causal for PID (LP/P)	7 (7%)	7 (7%)	0
Suspicious PID variants (LP/P)	8 (8%)	8 (8%)	0
Suspicious PID variants (VUS)	32 (34%)	28 (30%)	5 (5%)^*^
All PID related VUS, LP and P	47 (50%)	43 (46%)	5 (5%)^*^
Categories of the reanalysis responsible for the detection of genetic findings^†^			
Category 1 and 2: Variants detected in analysis for SNV or small indels	46 (49%)	42 (45%)	5 (5%)^*^
Category 3: Variants detected in analysis for SVs	1 (1%), ((2%))^‡^	1 (1%), ((2%))^‡^	0
Category 4^§^: SNV and SV VUS(s) in PID candidate genes and/or heterozygous in PID genes with AR inheritance	8 (8%)	—	8 (8%)
Incidental findings	4 (4%)	2 (2%)	2 (2%)

^*^1 of these 5 patients also had another SNV identified in the initial analysis. ^†^Categories of the reanalysis responsible for detection of the new finding: 1 = improved knowledge of genotype-phenotype, 2 = expansion of PID gene panel, 3 = SV analysis., or 4 = VUS(s) in candidate genes or heterozygous VUS(s) in genes with AR inheritance. ^‡^The one patient with a pathogenic SV is listed relative to the number of patients analysed for SVs (n = 56) in double brackets. ^§^Patients with VUS(s) in PID candidate genes and/or heterozygous VUS(s) in PID genes with AR inheritance detected in the reanalysis were described separately and thus not counted in Suspicious PID variants (VUS).

AR, Autosomal recessive; LP, likely pathogenic; P, Pathogenic; PID, Primary immune deficiency; SNV, Single nucleotide variant or small indel; SV, Structural variant; VUS, Variant of uncertain significance.

One patient with a causal variant identified with PID-1 was excluded from reanalysis and therefore not included in this table.

### Single Nucleotide Variation and Small Indels Analysis

When the genetic data was reanalysed, a new suspicious SNV VUS was identified in five patients (5%). The genetic variant details are shown in **Table 2**. We divided the patients into four groups based on the category of the reanalysis responsible for the detection of the new finding, as shown in **Table 3**. Category 1) Three of the patients (*19S, 49S, 59S*) had a new VUS added due to improved knowledge of phenotype-genotype correlation in a gene also included in *PID-1*. Category 2) Two patients (*29S, 31S*) had a VUS added due to the expansion of the PID gene panel. Additionally, one patient had an incidental finding (*7S*). Of these five patients with new PID related VUS(s), two patients (*19S* and *59S*) had a single VUS in an AR/autosomal dominant (AD) gene: *FLG* and *SERPING1*, respectively, and three patients (*29S, 31S, 49S*) had a single VUS in an AD gene: *OAS1*, *TNFAIP3* and *NFKB1*, respectively. These five patients’ phenotypes were compatible with the AR/AD or AD heritable disorder and functional immunological analysis in addition to genetic analysis of parents was recommended (where possible).

### Structural Variation Analysis

One patient had a pathogenic SV detected in the initial analysis (*63S*). Category 3) In the reanalysis we only detected SVs in either PID candidate genes and/or heterozygous in PID genes with autosomal recessive inheritance. These variants were included in Category 4 (below).

### SNV and SV VUS(s) in PID Candidate Genes and/or Heterozygous in PID Genes With Autosomal Recessive Inheritance

Category 4) We identified eight patients with SNV or SV VUS(s) in PID candidate genes (n=6) and/or heterozygous VUSs in PID genes with AR inheritance (n=2). Of the 56 individuals with WGS data, five patients (9%) had such a SV of uncertain significance identified (*10FP, 27S, 32S, 33S, 39FA1*). Four patients (*9S, 16S, 27S, 75S*) had a heterozygous VUS (SNV or SV) in a PID candidate gene. In one of these patients (*27S*) with Combined Immune Deficiency (CID), we detected a heterozygous, frameshift deletion in *LAG3* (lymphocyte-activation gene 3), which is involved in T-cell receptor signalling (32)*. LAG3* was not included in the *PID-2* gene panel. Further, one patient (*75S*) had a pathogenic incidental finding in addition to the new suspicious VUS in a PID candidate gene. In this patient, we detected a hemizygous pathogenic deletion in *EDA*, which was causal for the patient´s *hypohidrotic ectodermal dysplasia* phenotype. *EDA* is not included in the *PID-2* gene panel. The *EDA* variant was categorized as an incidental finding. Notably, during the initial WGS analysis of this patient, who also presented a phenotype of recurrent infections, genes related to ectodermal dysplasia were included in addition to the *PID-1* gene panel. SV analysis was not implemented at the time of the initial analysis. Furthermore, Array CGH had been performed which could not detect the deletion. Two patients (*33S, 39FA1*) had two VUSs in a PID candidate gene. These patients both had a SV and a SNV of uncertain significance in the same gene and potentially in trans (these SNVs were not included in the results of SNV analysis above). Two patients (*10FP, 32S*) had a single VUS in an autosomal recessive (AR) gene. These two patients both had a heterozygous tandem duplication in *DOCK8*. One of the patients presented a phenotype compatible with Hyper IgE syndrome. We added analysis for *DOCK8* deep intronic SNVs and small indels in these two cases due to prior reports of intron variants in patients with *DOCK8* deficiency. We did not identify LP or P variants in this analysis. Finally, one patient (*7S*) had an incidental finding (VUS) detected at reanalysis.

### Genetic Results Across ESID (2019) Subgroups

**Figure 2:**
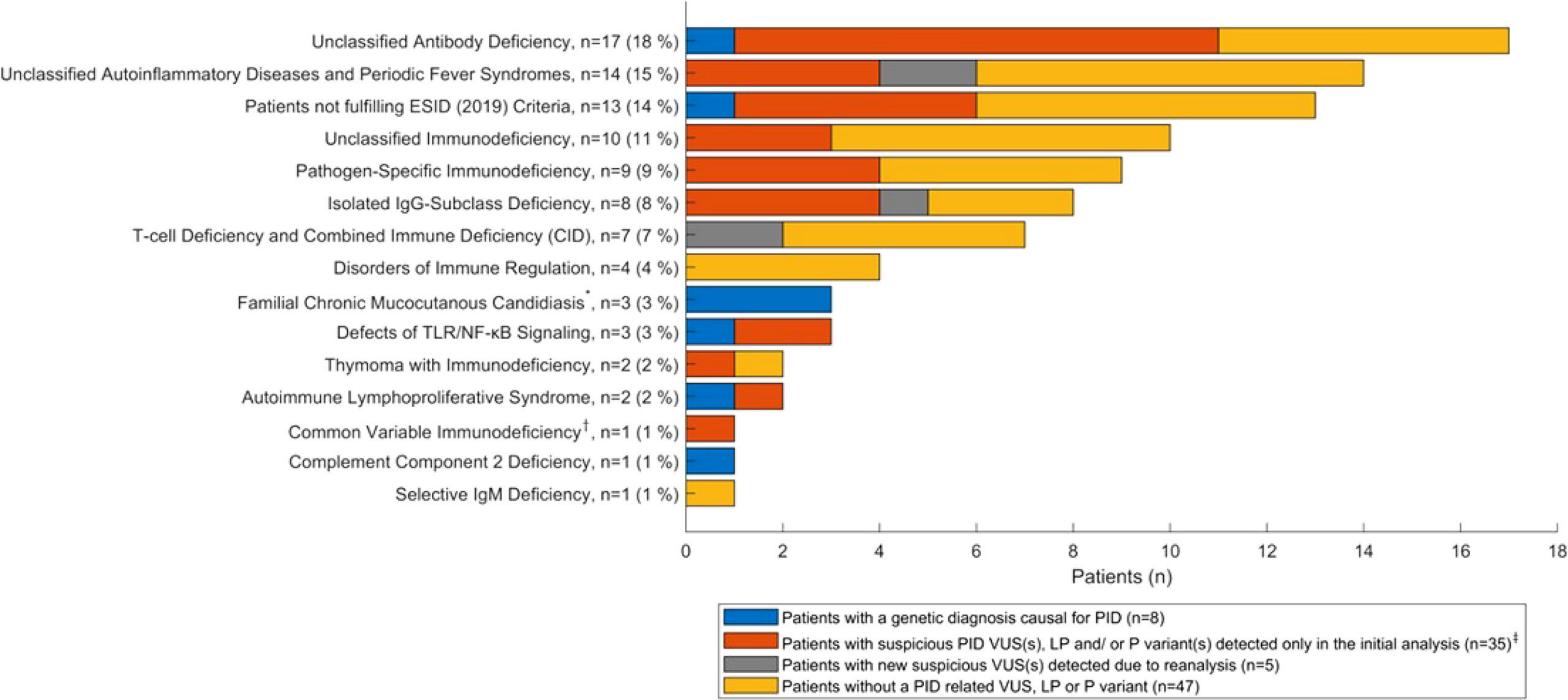
Distribution of the genetic results (PID-1 and PID-2 combined) across ESID (2019) subgroups (n = 95 patients). *The Familial Chronic Mucocutanous Candidiasis subgroup includes the one patient excluded from reanalysis. †One child with CVID was included, as the child is part of a family including a parent and sibling with antibody deficiency. ‡Includes eight patients with suspicious PID variants (LP/P) and 28 patients with suspicious PID variants (VUS), of which one is included in the five patients with new suspicious VUS(s) detected due to reanalysis, since this patient also had a suspicious PID VUS detected in the reanalysis. VUS, variant of uncertain significance; LP, likely pathogenic; P, pathogenic.

**Figure 2** shows the distribution of genetic results in patients across the different ESID (2019) (17) subgroups. In this figure, the results of the initial and reanalysis (*PID-1* and *PID-2*) are combined. Variants were identified in patients within most clinical subgroups.

### Re-Evaluation of Variants Identified in the Initial Analysis (*PID-1*)

All variants identified as part of the initial analysis with *PID-1* were re-evaluated. The results of specific immunologic analysis and/or segregation analysis in the patients with variants detected in the initial analysis (*PID-1*) are presented in **Supplementary Tables 2, 3**, where these analyses had been performed. This led to a re-classification in two patients with Familial Chronic Mucocutanous Candidiasis (CMC), ID *45S* and *46S*, who both had a VUS in *STAT1*, which were reclassified to P and LP variants, respectively. These two patients were part of the original 9 patients with a genetic diagnosis causal for the PID phenotype after the initial analysis (*PID-1*). ID *45S* had a heterozygous, gain of function variant (*STAT1*, c.1204_1205delinsTT, p.Ala402Phe). Subsequent analysis proved the variant to segregate with the phenotype in several family members. Additionally, functional immunological analysis confirmed the effect. Therefore, this variant was reclassified to a P variant. ID *46S* had a heterozygous, missense variant (*STAT1*, c.823C>A, p.Gln275Lys), which was absent from controls. Subsequent analysis of the parents proved the variant to be *de novo*. Therefore, the variant was reclassified to a LP variant. We did not re-classify any VUSs to benign or likely benign variants.

## Discussion

This study was conducted to investigate the value of performing a reanalysis of WES/WGS data in a PID population consisting of patients over 16 years of age, after an inclusion period of approximately 4 ½ years. The re-analysis (*PID-2*) was performed a median of 23 months after the initial analysis (*PID-1*). Upon re-analysis new suspicious PID VUSs were detected in 5% of the patients. Since all new PID related variants (SNVs and SVs) were VUSs, the number of causal genetic PID diagnoses did not increase after the reanalysis. The total rate of patients having PID VUS(s), LP or P variant(s) increased by 4% to a total rate of 50%. Three patients had a VUS identified due to improved knowledge of genotype-phenotype interaction and two patients had a VUS in new PID genes added in *PID-2*. In addition, we identified five patients with new SVs of uncertain significance and three patients with SNVs of uncertain significance. These eight patients were described separately, as they have VUS(s) in either PID candidate genes or heterozygous in established PID genes with AR inheritance. Reasons for the identification of new variants include the updated tools for calling of SNVs/small indels and SVs as well as improvements of *in silico* analysis. Our findings indicate a possible diagnostic importance of analysing SVs in an adult PID population. Therefore, we aim to transfer the genetic analysis from WES to WGS to be able to perform SV analysis in selected cases with high suspicion of a PID. Furthermore, in selected cases presenting symptoms beyond a PID phenotype, a re-evaluation of the indication for adding additional gene panels or extending to full exome/genome analysis should be considered. The detection of VUSs in new PID candidate genes together with functional immunological analysis of affected pathway(s) can potentially improve the knowledge of genotype-phenotype correlations underlying PIDs.

When comparing rates of genetic findings between PID cohorts, the selection of patients is crucial. Including patients with more severe phenotypes, patients with symptom onset in childhood and a positive family history will lead to a higher molecular diagnostic rate (33). Further, a proportion of PIDs with symptom onset in adulthood are suspected to be caused by polygenetic (34, 35) and/or multifactorial inheritance (36). Additional knowledge of mono- and polygenic variation underlying these PIDs remains to be gained. Methods beyond analysis of single cases and smaller cohorts are needed to detect new genetic variants contributing to PID phenotypes (37, 38). In one relatively small cohort of six PID patients analysis of WGS data, including copy number variation (deletions and duplications), led to the detection of explanatory variants in all cases analysed (39).

Unclassified antibody deficiencies and unclassified autoinflammatory diseases constitute the major clinical subgroups in our PID cohort (**Table 2**). The proportion of antibody deficiencies corresponds to the proportion in a large study of primarily adult PID patients (6). Furthermore, the proportion consisting of CIDs was relatively low (7%), whereas the proportion of pathogen specific immunodeficiencies was relatively high (9%). These deviations could be due to a larger proportion of children in the study by Smith et al. However, these subgroups contain small numbers of patients. Notably, in the subgroup of patients, who did not fulfil ESID clinical criteria, a variant was identified in six of the 13 patients, of which one had a homozygous, pathogenic variant in *ERAP1* (*74T*) and five had VUS(s) in PID genes. In addition, three patients had familial chronic mucocutaneous candidiasis (CMC) due to pathogenic gain of function variants in *STAT1*.

Two studies from the UK that investigated a semiautomated reanalysis of WES data in more than 1000 families with developmental delay (13) and more than 2000 consecutive cases of WES (12), showed increases of molecular diagnostic rates of 13% (27 to 40%) and 11.5% (25 to 36%), respectively. However, different disease groups are clearly not directly comparable. The two studies both included children and the molecular diagnostic rates did not include variants of uncertain significance. In contrast, our findings of new variants in PID genes were solely VUSs. Notably, our cohort primarily investigated adult patients and few children included in families with a suspected PID. As well as developmental delay, PID phenotypes are very heterogenous, especially if milder phenotypes of PID are included. Also, PID patients can present with a symptom onset in adulthood, making it more difficult to determine the impact and pathogenicity of genetic variants, which may be inherited from an apparently unaffected parent. Finally, both PID and developmental delay can potentially arise due to variants with variable expression and/or reduced penetrance.

Data analyses and variant classifications were performed by six molecular biologists and clinical geneticists during the 5-year study period. To limit the effect of interobserver variability, all interpreters followed the ACMG international guidelines (29), and all findings were confirmed by two interpreters. However, inevitably there will be interobserver variability of variant interpretation, which can affect the rate of PID related variants identified. Additionally, when rates of genetic variants are compared between PID cohorts, there can be inter-laboratory variability. Further, over time some monogenic PID disorders known to be caused by autosomal recessive inheritance have been proven also to be caused by autosomal dominant inheritance (40). Therefore, during this study period, the number of heterozygous variants reported has increased. Finally, more patients were included as trio analysis in the beginning of the inclusion period, and more singletons were included towards the end. Trio analysis are often preferable for WES/WGS data interpretation since trio analyses can provide information on potential *de novo* variants or compound heterozygosity for two variants in the same gene. Therefore, the change to a predominant inclusion of singletons later in the inclusion period may also have led to an increase of heterozygous variants reported, where subsequent analysis of parents is needed to unravel *de novo* variants or compound heterozygosity for two variants.

One of the limitations of the study was, that analysis for low level of germline mosaicism (<20% of reads) was not performed, since WGS analysis is not ideal for identifying potential germline mosaicism. Another limitation of our study was in the analysis of genes known to have pseudogenes, e.g. *IKBKG, NCF1, C4A*, and *C4B*. For such regions specialized bioinformatics pipelines could be required to accurately call the variants in these genes, which was not applied in our study. This limitation is included as a disclaimer in the clinical reports. The targeted analysis performed in our study could also be considered as a limitation, since additional VUSs in new PID candidate genes may be detected by performing analysis of the entire exome or genome. Another limitation was, that we did not consider the deep intronic variants (located deeper then +/-20bp to the exons). However, as the performance of any kind of functional analysis for potential effect of splice- or other non-coding variants was not within our study scope, we decided to focus our analysis to the +/-20bp exon-flanking regions in order to simplify the clinical interpretation of the variants.

Performing a reanalysis of broad sequencing data is resource demanding. As such, a potential gain in diagnostic performance after data reanalysis needs to be balanced against the time and cost involved (41). In contrast to other studies including automated reanalysis, we did not detect new genetic diagnoses upon reanalysis. Yet our results showed that performing the reanalysis may improve genetic results in prior undiagnosed patients. Our findings included both SNVs and SVs in new PID genes (added in *PID-2*), which shows the importance of regularly extending gene panels for reanalysis in PID. The *PID-2* gene panel contains many PID candidate genes (newly described in relation to PID) without prior reports in the literature of variants in patients. Therefore, until the knowledge of gene-disease interaction improves, variants identified in PID candidate genes within *PID-2* will primarily be classified as VUSs. Within the Genomics England Panel App approximately half of the genes in the current PID gene panel (Primary immune deficiency Version 2.498) are categorized as red (not enough evidence) or amber (moderate evidence of gene-disease association), where additional evidence is needed to confirm the genotype-phenotype correlation. Thus, additional evidence of pathogenicity for the VUSs identified in our PID cohort may arise from individual, specific functional immunological analysis and segregation analysis.

Due to the high level of heterogeneity of PIDs, the interpretation of rare variants and planning of functional analysis, particularly of variants in PID candidate genes, can benefit from international collaborations, e.g. GeneMatcher (42). Due to costs of manual reanalysis an automated reanalysis of VUS and LP variants incorporating genomic, phenotypic, and pedigree data could ideally prioritize the variants in established PID genes for clinicians to review, in line with what has been implemented for other disease groups (43–46). Currently, new methods are being developed and implemented to improve automation of genetic reanalysis (8, 10, 47). In PID populations, where most genetic variants are VUSs, patients could benefit from regular semiautomated reanalysis of WES/WGS data with a reduced demand of time for manual variant interpretation. However, due to the large extent of PID candidate genes, VUSs detected by automated reanalysis methods in these genes will require additional evidence of pathogenicity from segregation studies, functional analysis and/or other reports of cases with similar phenotypes.

In conclusion, our reanalysis of WES/WGS data did not lead to novel genetic diagnoses causal for PID. We detected new suspicious PID VUS(s) in five (5%) patients suspected of PID. Additionally, eight patients had SNV/SV VUS(s) in PID candidate genes or heterozygous VUS(s) PID genes with AR inheritance. Our data indicate a possible diagnostic value of reassessing WES/WGS data with an extended gene panel and performing analysis of SV in a PID population of primarily adults. To our knowledge, this is the first study to investigate the impact of a WES/WGS data reanalysis and to include structural variation analysis in a PID population. Future improved knowledge of genotype-phenotype correlation in PID candidate genes may provide additional certainty of the effect of the identified VUSs.

## Data Availability Statement

The datasets presented in this article are not readily available because of national legislation. Requests to access the datasets should be directed to sara.bohnstedt.moerup@regionh.dk.

## Ethics Statement

The patients/participants legal guardian/next of kin provided written informed consent to participate in this study. The study was reviewed and approved by the Danish Regional Ethics Committee (H-15009663).

## Author Contributions

HM, RM, TK, and JL: Planned the study. SM, JR, HM, JL, DM, MH, LB, and TK: Contributed to the design. SM, TK, TM, and MH: Acquisition of the phenotype data. SM, MG, LN-P, RM, and LB: Interpretation, analyses, and quality control of genetic data. SM, HM, and HH: Interpretation of immunologic data. SM, JR, DM, MH, and LB: Wrote the manuscript. All authors critically reviewed and approved the final version of the manuscript.

## Funding

This work is funded by a grant from the Danish National Research Foundation (DNRF126).

## Conflict of Interest

TK has participated in advisory boards for Gilead Sciences, GlaxoSmithKline/ViiV and MSD. TK has received fees for teaching from Takeda and CSL Behring, and a research grant from Gilead Sciences. MH has participated in advisory boards for AstraZeneca, Gilead Sciences, GlaxoSmithKline/ViiV, MSD, Roche, and Sobi. MH has received fees for teaching from Gilead Sciences and GSK.

The remaining authors declare that the research was conducted in the absence of any commercial or financial relationships that could be construed as a potential conflict of interest.

## Publisher’s Note

All claims expressed in this article are solely those of the authors and do not necessarily represent those of their affiliated organizations, or those of the publisher, the editors and the reviewers. Any product that may be evaluated in this article, or claim that may be made by its manufacturer, is not guaranteed or endorsed by the publisher.

## References

[1] Mahlaoui N Warnatz K Jones A Workman S Cant A. Advances in the Care of Primary Immunodeficiencies (PIDs): From Birth to Adulthood. J Clin Immunol (2017) 37:452–60. doi: 10.1007/s10875-017-0401-y PMC5489581 28523402

[2] Zhang Q Frange P Blanche S Casanova JL. Pathogenesis of Infections in HIV-Infected Individuals: Insights From Primary Immunodeficiencies. Curr Opin Immunol (2017) 48:122–33. doi: 10.1016/j.coi.2017.09.002 PMC5682227 28992464

[3] Denning L. Primary Immunodeficiencies for General Practitioners – Making a Difference in Diagnosing Severe Illness. J Fam Med Prim Care (2017) 6:709. doi: 10.4103/jfmpc.jfmpc_414_16 PMC5848383 29564248

[4] Tangye SG Al-Herz W Bousfiha A Chatila T Cunningham-Rundles C Etzioni A. Human Inborn Errors of Immunity: 2019 Update on the Classification From the International Union of Immunological Societies Expert Committee. J Clin Immunol (2020) 40:24–64. doi: 10.1007/s10875-019-00737-x 31953710 PMC7082301

[5] Yska HAF Elsink K Kuijpers TW Frederix GWJ van Gijn ME van Montfrans JM. Diagnostic Yield of Next Generation Sequencing in Genetically Undiagnosed Patients With Primary Immunodeficiencies: A Systematic Review. J Clin Immunol (2019) 39:577–91. doi: 10.1007/s10875-019-00656-x PMC6697711 31250335

[6] Thaventhiran JED Lango H Burren OS Rae W Greene D Staples E. Whole-Genome Sequencing of a Sporadic Primary Immunodeficiency Cohort. Nature (2020) 10:90–5. doi: 10.1038/s41586-020-2265-1 PMC7334047 32499645

[7] Wenger AM Guturu H Bernstein JA Bejerano G. Systematic Reanalysis of Clinical Exome Data Yields Additional Diagnoses: Implications for Providers. Genet Med (2017) 19:209–14. doi: 10.1038/gim.2016.88 27441994

[8] Baker SW Murrell JR Nesbitt AI Pechter KB Balciuniene J Zhao X. Automated Clinical Exome Reanalysis Reveals Novel Diagnoses. J Mol Diagn (2019) 21:38–48. doi: 10.1016/j.jmoldx.2018.07.008 30577886

[9] Salfati EL Spencer EG Topol SE Muse ED Rueda M Lucas JR. Re-Analysis of Whole-Exome Sequencing Data Uncovers Novel Diagnostic Variants and Improves Molecular Diagnostic Yields for Sudden Death and Idiopathic Diseases. Genome Med (2019) 11:1–8. doi: 10.1186/s13073-019-0702-2 31847883 PMC6916453

[10] Seo GH Lee H Lee J Han H Cho YK Kim M. Diagnostic Performance of Automated, Streamlined, Daily Updated Exome Analysis in Patients With Neurodevelopmental Delay. Mol Med (2022) 28. doi: 10.1186/s10020-022-00464-x PMC8962085 35346031

[11] Costain G Jobling R Walker S Reuter MS Snell M Bowdin S. Periodic Reanalysis of Whole-Genome Sequencing Data Enhances the Diagnostic Advantage Over Standard Clinical Genetic Testing. Eur J Hum Genet (2018) 26:740–4. doi: 10.1038/s41431-018-0114-6 PMC5945683 29453418

[12] Liu P Lupski JR Yang Y. Reanalysis of Clinical Exome Sequencing Data. New Engl J Med (2020) 26:740–744. doi: 10.1056/NEJMc1812033 PMC6934160 31216405

[13] Wright CF McRae JF Clayton S Gallone G Aitken S FitzGerald TW. Making New Genetic Diagnoses With Old Data: Iterative Reanalysis and Reporting From Genome-Wide Data in 1,133 Families With Developmental Disorders. Genet Med (2018) 20:1216–23. doi: 10.1038/gim.2017.246 PMC5912505 29323667

[14] Westh L Mogensen TH Dalgaard LS Bernth Jensen JM Katzenstein T Hansen ABE. Identification and Characterization of a Nationwide Danish Adult Common Variable Immunodeficiency Cohort. Scand J Immunol (2017) 85:450–61. doi: 10.1111/sji.12551 28370285

[15] Ameratunga R Woon ST. Perspective: Evolving Concepts in the Diagnosis and Understanding of Common Variable Immunodeficiency Disorders (CVID). Clin Rev Allergy Immunol (2020) 59:109–21. doi: 10.1007/s12016-019-08765-6 31720921

[16] Human Phenotype Ontology. Available at: https://hpo.jax.org/app/.

[17] Seidel MG Kindle G Gathmann B Quinti I Buckland M van Montfrans J. The European Society for Immunodeficiencies (ESID) Registry Working Definitions for the Clinical Diagnosis of Inborn Errors of Immunity. J Allergy Clin Immunol Pract (2019) 7:1763–70. doi: 10.1016/j.jaip.2019.02.004 30776527

[18] Bonilla FA Khan DA Ballas ZK Chinen J Frank MM Hsu JT. Practice Parameter for the Diagnosis and Management of Primary Immunodeficiency. J Allergy Clin Immunol (2014) 136:1186–1205.e78. doi: 10.1016/j.jaci.2015.04.049 26371839

[19] Genomics England PanelApp. Available at: https://panelapp.genomicsengland.co.uk/.

[20] McKenna A Hanna M Banks E Sivachenko A Cibulskis K Kernytsky A. The Genome Analysis Toolkit: A MapReduce Framework for Analyzing Next-Generation DNA Sequencing Data. Genome Res (2010) 20:1297–303. doi: 10.1101/gr.107524.110 PMC2928508 20644199

[21] Chen X Schulz-Trieglaff O Shaw R Barnes B Schlesinger F Källberg M. Manta: Rapid Detection of Structural Variants and Indels for Germline and Cancer Sequencing Applications. Bioinformatics (2016) 32:1220–2. doi: 10.1093/bioinformatics/btv710 26647377

[22] Layer RM Chiang C Quinlan AR Hall IM. LUMPY: A Probabilistic Framework for Structural Variant Discovery. Genome Biol (2014) 15:1–19. doi: 10.1186/gb-2014-15-6-r84 PMC4197822 24970577

[23] Abyzov A Urban AE Snyder M Gerstein M. CNVnator: An Approach to Discover, Genotype, and Characterize Typical and Atypical CNVs From Family and Population Genome Sequencing. Genome Res (2011) 21:974–84. doi: 10.1101/gr.114876.110 PMC3106330 21324876

[24] Thorvaldsdóttir H Robinson JT Mesirov JP. Integrative Genomics Viewer (IGV): High-Performance Genomics Data Visualization and Exploration. Brief Bioinform (2013) 14:178–92. doi: 10.1093/bib/bbs017 PMC3603213 22517427

[25] Zhang P Bigio B Rapaport F Zhang SY Casanova JL Abel L. PopViz: A Webserver for Visualizing Minor Allele Frequencies and Damage Prediction Scores of Human Genetic Variations. Bioinformatics (2018) 34:4307–9. doi: 10.1093/bioinformatics/bty536 PMC6289133 30535305

[26] The Human Gene Mutation Database. Available at: http://www.hgmd.cf.ac.uk/ac/index.php.

[27] ClinVar. Available at: https://www.ncbi.nlm.nih.gov/clinvar/.

[28] Shamsani J Kazakoff SH Armean IM McLaren W Parsons MT Thompson BA. A Plugin for the Ensembl Variant Effect Predictor That Uses MaxEntScan to Predict Variant Spliceogenicity. Bioinformatics (2019) 35:2315–7. doi: 10.1093/bioinformatics/bty960 PMC6596880 30475984

[29] Richards S Aziz N Bale S Bick D Das S Gastier-Foster J. Standards and Guidelines for the Interpretation of Sequence Variants: A Joint Consensus Recommendation of the American College of Medical Genetics and Genomics and the Association for Molecular Pathology. Genet Med (2015) 17:405–24. doi: 10.1038/gim.2015.30 PMC4544753 25741868

[30] Deignan JL Chung WK Kearney HM Monaghan KG Rehder CW Chao EC. Points to Consider in the Reevaluation and Reanalysis of Genomic Test Results: A Statement of the American College of Medical Genetics and Genomics (ACMG). Genet Med (2019) 21:1267–70. doi: 10.1038/s41436-019-0478-1 PMC6559819 31015575

[31] Richardson AM Moyer AM Hasadsri L Abraham RS. Diagnostic Tools for Inborn Errors of Human Immunity (Primary Immunodeficiencies and Immune Dysregulatory Diseases). Curr Allergy Asthma Rep (2018) 18. doi: 10.1007/s11882-018-0770-1 29470720

[32] Maruhashi T Okazaki IM Sugiura D Takahashi S Maeda TK Shimizu?A3B2 show [#,32] ?> K. LAG-3 Inhibits the Activation of CD4 + T Cells That Recognize Stable pMHCII Through its Conformation-Dependent Recognition of pMHCII. Nat Immunol (2018) 19:1415–26. doi: 10.1038/s41590-018-0217-9 30349037

[33] Sogkas G Dubrowinskaja N Schütz K Steinbrück L Götting J Schwerk N. Diagnostic Yield and Therapeutic Consequences of Targeted Next-Generation Sequencing in Sporadic Primary Immunodeficiency. Int Arch Allergy Immunol (2021) 1–13. doi: 10.1159/000519199 PMC8985027 34619682

[34] Amaya-Uribe L Rojas M Azizi G Anaya JM Gershwin ME. Primary Immunodeficiency and Autoimmunity: A Comprehensive Review. J Autoimmun (2019) 99:52–72. doi: 10.1016/j.jaut.2019.01.011 30795880

[35] Grimbacher B Warnatz K Yong PFK Korganow AS Peter HH. The Crossroads of Autoimmunity and Immunodeficiency: Lessons From Polygenic Traits and Monogenic Defects. J Allergy Clin Immunol (2016) 137:3–17. doi: 10.1016/j.jaci.2015.11.004 26768758

[36] Azizi G Yazdani R Rae W Abolhassani H Rojas M Aghamohammadi A. Monogenic Polyautoimmunity in Primary Immunodeficiency Diseases. Autoimmun Rev (2018) 17:1028–39. doi: 10.1016/j.autrev.2018.05.001 30107266

[37] Itan Y Casanova JL. Novel Primary Immunodeficiency Candidate Genes Predicted by the Human Gene Connectome. Front Immunol (2015) 6:142. doi: 10.3389/fimmu.2015.00142 25883595 PMC4381650

[38] Kwok AJ Mentzer A Knight JC. Host Genetics and Infectious Disease: New Tools, Insights and Translational Opportunities. Nat Rev Genet (2021) 22:137–53. doi: 10.1038/s41576-020-00297-6 PMC7716795 33277640

[39] Mousallem T Urban TJ McSweeney KM Kleinstein SE Zhu M Adeli M. Clinical Application of Whole-Genome Sequencing in Patients With Primary Immunodeficiency. J Allergy Clin Immunol (2015) 136:476–479.e6. doi: 10.1016/j.jaci.2015.02.040 25981738 PMC5037571

[40] Procopio V Manti S Bianco G Conti G Romeo A Maimone F. Genotype-Phenotype Correlation in FMF Patients: A “Non Classic” Recessive Autosomal or “Atypical” Dominant Autosomal Inheritance? Gene (2018) 641:279–86. doi: 10.1016/j.gene.2017.10.068 29080837

[41] Schwarze K Buchanan J Fermont JM Dreau H Tilley MW Taylor JM. The Complete Costs of Genome Sequencing: A Microcosting Study in Cancer and Rare Diseases From a Single Center in the United Kingdom. Genet Med (2020) 22:85–94. doi: 10.1038/s41436-019-0618-7 31358947 PMC6944636

[42] GeneMatcher. Available at: https://genematcher.org/.

[43] Matalonga L Hernández-Ferrer C Piscia D Cohen E Cuesta I Danis D. Solving Patients With Rare Diseases Through Programmatic Reanalysis of Genome-Phenome Data. Eur J Hum Genet (2021) 29:1337–47. doi: 10.1038/s41431-021-00852-7 PMC8440686 34075210

[44] Ji J Leung ML Baker S Deignan JL Santani A. Clinical Exome Reanalysis: Current Practice and Beyond. Mol Diagnosis Ther (2021) 25:529–36. doi: 10.1007/s40291-021-00541-7 PMC8410709 34283395

[45] Thompson R Papakonstantinou Ntalis A Beltran S Töpf A de Paula Estephan E Polavarapu K. Increasing Phenotypic Annotation Improves the Diagnostic Rate of Exome Sequencing in a Rare Neuromuscular Disorder. Hum Mutat (2019) 40:1797–812. doi: 10.1002/humu.23792 31231902

[46] Shashi V Schoch K Spillmann R Cope H Tan QKG Walley N. A Comprehensive Iterative Approach is Highly Effective in Diagnosing Individuals Who are Exome Negative. Genet Med (2019) 21:161–72. doi: 10.1038/s41436-018-0044-2 PMC6295275 29907797

[47] O’Brien TD Campbell NE Potter AB Letaw JH Kulkarni A Richards CS. Artificial Intelligence (AI)-Assisted Exome Reanalysis Greatly Aids in the Identification of New Positive Cases and Reduces Analysis Time in a Clinical Diagnostic Laboratory. Genet Med (2022) 24:192–200. doi: 10.1016/j.gim.2021.09.007 34906498

[48] Mørk N Kofod-Olsen E Sørensen KB Bach E Ørntoft TF Østergaard L. Mutations in the TLR3 Signaling Pathway and Beyond in Adult Patients With Herpes Simplex Encephalitis. Genes Immun (2015) 16:552–66. doi: 10.1038/gene.2015.46 26513235

[49] Nijmegen PID Panel DG 2.5. Available at: https://www.radboudumc.nl/getmedia/eecc00c0-6ff7-4f4e-8423-e78e1ef3b2bc/PRIMARY-IMMUNODEFICIENCIES-DG25DG26.aspx.

[50] Nijmegen PID Panel DG2.18. Available at: https://www.radboudumc.nl/getmedia/f7b000f4-2332-42e6-8dfd-5519d346aa85/PRIMARYIMMUNODEFICIENCY_DG218.aspx.

[51] Tangye SG Al-Herz W Bousfiha A Cunningham-Rundles C Franco JL Holland SM. The Ever-Increasing Array of Novel Inborn Errors of Immunity: An Interim Update by the IUIS Committee. J Clin Immunol (2021) 41:666–79. doi: 10.1007/s10875-021-00980-1 PMC7889474 33598806

