# Supplementary material for: Added Value of Reanalysis of Whole Exome- and Whole Genome Sequencing Data From Patients Suspected of Primary Immune Deficiency Using an Extended Gene Panel and Structural Variation Calling
Source: Front Immunol. 2022 Jun 30;13:906328. doi: 10.3389/fimmu.2022.906328 (PMC9302041; doi:10.3389/fimmu.2022.906328)
Supplement: Supplementary file 1 [file DataSheet_1.docx]

**Supplementary**

**References in *PID-1***

References included in the development of *PID-1* (number of unique PID genes in panel):

- *2016 European Society for Immunodeficiencies online registry of diseases genes* (n=252). 2016 version is no longer accessible online.
- *Veritas genetics immune disorders list,* (n=136). No longer accessible online.
- *Practice parameter for the diagnosis and management of primary immunodeficiency*, Bonilla, et al.(18), Table 2 (n=246).
- Mørk et al.(48), online Supplementary information (n=204)
- *Nijmegen Genetics* PID panel, DG 2.5(49) (n=301)
- Miscellaneous genes within individual references (n=17)
- Local PID gene lists within the Department of Immunology, Rigshospitalet (n=270) and the Department of Infectious Diseases, Aarhus University Hospital, Skejby (n=245).

**References added in *PID-2***

*PID-2* was developed by comparing new PID gene panels with *PID-1*. Additionally, we evaluated the genes within the 2015 gene panel, which led to the exclusion of 19 genes due to 1) 3 were genetic loci (*SLEB13, SLEB7, HPLH1*) 2) 10 genes were renamed due to updated HGNC nomenclature 3) 1 gene (*PTPN11*) was excluded due to being present in Nijmegen DG 2.5 gene panel and removed from the updated DG2.18 version 4) HGNC IDs for 3 genes were not in *BioMart* (*IGH, IGK and SF3B6*) and 5) 2 genes were not included in hg19 (*SLEB3 and TRBC1*).

References included in the development of *PID-2* (number of unique PID genes added when comparing with PID-1 and above added PID gene panel):

- PID gene panels available within the Genomics England PanelApp(19) (*Immunological disorders SuperPanel (Version 0.803), Primary immunodeficiency panel (Version 2.369),* *Periodic fever syndromes panel (Version 1.12) and Viral Resistance panel (Version 0.63))* (n=184 genes to add).
- *The International Union of Immunological Societies Expert Committee´s 2019 Classification*(4), tables 1-9 (n=8 genes to add).
- Updated *Nijmegen Genetics PID panel*, DG2.18(50) (n=8 genes to add).
- *Interim update by the IUIS Committee Tangye et al. 2020 (submitted at the time of the development of PID-2)* (n=12 genes to add). (In the publication(51) 8/12 genes added in PID-2 were removed).
- 3 miscellaneous genes were added during the review (*IGLC1, IGHG1 and IGHE).*

**Table 1: List of 665 genes in *PID-2.***

Genes added in *PID-2* are in bold.

| ***ABI3*** | ***BACH2*** | *CARD14* | *CD81* | *CORO1A* | *DNMT3B* | ***FBF1*** | ***GIMAP6*** | *IFNL3* | *IL21R* | *JAGN1* |
| --- | --- | --- | --- | --- | --- | --- | --- | --- | --- | --- |
| ***ACD*** | *BANK1* | ***CARD8*** | *CD8A* | *CR2* | *DOCK2* | ***FBRS*** | ***GINS1*** | *IFNL4* | ***IL22*** | ***JAK1*** |
| ***ACKR1*** | ***BCL10*** | *CARD9* | ***CDC42*** | *CREBBP* | *DOCK8* | *FCER1G* | *GJC2* | ***IGHE*** | *IL23A* | *JAK2* |
| *ACP5* | *BCL11B* | ***CARMIL2*** | *CDCA7* | *CSF1R* | *DPP10* | *FCGR1A* | ***GRHL2*** | ***IGHG1*** | ***IL23R*** | *JAK3* |
| *ACTB* | *BLK* | *CASP1* | ***CDH17*** | *CSF2RA* | ***DPP4*** | *FCGR2A* | ***GSN*** | ***IGHG2*** | *IL2RA* | ***KDM6A*** |
| *ADA* | *BLM* | *CASP10* | *CDKN2B* | *CSF2RB* | *DSG1* | *FCGR2B* | *GTF2H5* | *IGHM* | ***IL2RB*** | ***KIR3DL1*** |
| ***ADA2*** | *BLNK* | *CASP8* | *CEBPE* | *CSF3R* | ***EFL1*** | *FCGR3A* | ***GUCY2C*** | ***IGKC*** | *IL2RG* | ***KMT2A*** |
| *ADAM17* | *BLOC1S6* | ***CAVIN1*** | *CEBPG* | ***CST3*** | *ELANE* | *FCGR3B* | ***HAVCR2*** | ***IGLC1*** | ***IL31RA*** | *KMT2D* |
| *ADAR* | ***BPIFA1*** | ***CCBE1*** | *CFD* | *CTC1* | *ELF4* | *FCGRT* | *HAX1* | *IGLL1* | *IL36RN* | *KRAS* |
| *ADRB2* | ***BRCA1*** | *CCL11* | *CFH* | *CTLA4* | ***EPCAM*** | ***FCHO1*** | *HELLS* | *IKBKB* | ***IL4R*** | ***LACC1*** |
| *AGA* | *BRCA2* | ***CCL2*** | *CFHR1* | ***CTNNBL1*** | *EPG5* | *FCN3* | ***HLA-B*** | *IKBKE* | ***IL6*** | *LAMTOR2* |
| *AICDA* | *BRIP1* | ***CCL3*** | *CFHR2* | *CTPS1* | *ERAP1* | ***FERMT1*** | *HLA-C* | *IKBKG* | ***IL6R*** | ***LAT*** |
| *AIM2* | *BTK* | *CCL8* | *CFHR3* | *CTSC* | ***ERBIN*** | *FERMT3* | ***HLA-DQB1*** | *IKZF1* | ***IL6ST*** | *LCK* |
| *AIRE* | *C1QA* | *CCR5* | *CFHR4* | ***CXCL12*** | *ERCC2* | ***FGA*** | *HLA-DRB1* | *IKZF3* | ***IL7*** | ***LDLR*** |
| *AK2* | *C1QB* | *CD19* | *CFHR5* | ***CXCR1*** | *ERCC3* | *FLG* | ***HMOX1*** | *IL10* | *IL7R* | *LIG1* |
| *ALG13* | *C1QC* | *CD209* | *CFI* | *CXCR4* | ***ERCC4*** | ***FNIP1*** | *HNMT* | *IL10RA* | ***INO80*** | *LIG4* |
| *ALOX5* | *C1R* | *CD244* | *CFP* | *CYBA* | ***ERCC6L2*** | ***FOXM1*** | *HPS1* | *IL10RB* | *INSR* | *LPIN2* |
| ***ALPI*** | *C1S* | *CD247* | *CFTR* | *CYBB* | ***EXTL3*** | *FOXN1* | *HPS4* | *IL12A* | ***IRAK1*** | *LRBA* |
| ***ALPK1*** | *C2* | *CD27* | *CGAS* | ***CYBC1*** | *F12* | *FOXP3* | *HPS6* | *IL12B* | *IRAK4* | *LRRC8A* |
| *AP1S3* | *C3* | *CD28* | *CHD7* | ***DBR1*** | *FADD* | *FPR1* | ***HTR1A*** | *IL12RB1* | *IRF2BP2* | *LTA* |
| *AP3B1* | *sd* | *CD3D* | *CHUK* | ***DCLRE1B*** | *FANCA* | ***FPR2*** | ***HTRA2*** | ***IL12RB2*** | *IRF3* | *LTBP3* |
| ***AP3D1*** | *C4B* | *CD3E* | ***CIB1*** | *DCLRE1C* | *FANCB* | ***FPR3*** | ***HYOU1*** | *IL13* | ***IRF4*** | *LTBR* |
| ***APOA1*** | *C4BPA* | *CD3G* | *CIITA* | *DDX11* | *FANCC* | ***FUT2*** | *IBTK* | ***IL17A*** | *IRF5* | *LYST* |
| ***APOA2*** | *C4BPB* | *CD4* | *CLCN7* | *DDX41* | *FANCD2* | ***FAAP24*** | *ICOS* | *IL17F* | *IRF7* | ***LYZ*** |
| ***APOC2*** | *C5* | *CD40* | *CLEC4D* | *DDX58* | *FANCE* | *G6PC1* | ***ICOSLG*** | *IL17RA* | *IRF8* | ***MAD2L2*** |
| ***APOC3*** | *C6* | *CD40LG* | ***CLEC4M*** | ***DEF6*** | *FANCF* | *G6PC3* | *IFI16* | *IL17RB* | ***IRF9*** | *MAGT1* |
| *APOL1* | *C7* | *CD46* | *CLEC6A* | *DHFR* | *FANCG* | *G6PD* | *IFIH1* | ***IL17RC*** | *ISG15* | *MAL* |
| ***APP*** | *C8A* | ***CD48*** | *CLEC7A* | ***DIPK2B*** | *FANCI* | ***GAD1*** | *IFITM3* | *IL18* | *ITCH* | ***MAL2*** |
| ***ARHGEF1*** | *C8B* | *CD55* | ***CLPB*** | *DKC1* | *FANCL* | ***GATA1*** | ***IFNAR1*** | ***IL18BP*** | *ITGAM* | *MALT1* |
| ***ARPC1B*** | *C8G* | *CD59* | *CNBP* | ***DNAJC21*** | *FANCM* | *GATA2* | ***IFNAR2*** | *IL1RN* | *ITGB2* | *MAN2B1* |
| *ATM* | *C9* | ***CD70*** | ***COL7A1*** | *DNASE1* | *FAS* | ***GATA3*** | *IFNG* | *IL2* | *ITK* | ***MAN2B2*** |
| ***ATP6AP1*** | *CA2* | *CD79A* | *COLEC11* | *DNASE1L3* | *FASLG* | *GFI1* | *IFNGR1* | *IL20RA* | ***ITPKB*** | *MANBA* |
| ***B2M*** | *CARD11* | *CD79B* | *COPA* | ***DNASE2*** | ***FAT4*** | ***GIMAP5*** | *IFNGR2* | *IL21* | ***IVNS1ABP*** | ***MAP3K14*** |

| *MAP3K7* | *NBN* | ***OTULIN*** | ***POLR3F*** | *RECQL4* | ***SEC61A1*** | ***SRP54*** | *THBD* | *TRAF3IP2* | *ZBTB24* |
| --- | --- | --- | --- | --- | --- | --- | --- | --- | --- |
| ***MAPK8*** | *NCF1* | *PADI4* | ***POMP*** | ***REL*** | ***SELPLG*** | ***SRP72*** | *TICAM1* | *TRAF6* | ***ZC3HC1*** |
| *MASP1* | *NCF2* | *PALB2* | ***POT1*** | ***RELA*** | *SEMA3E* | *STAT1* | *TINF2* | *TREX1* | ***ZFP36*** |
| *MASP2* | *NCF4* | *PARN* | *PRF1* | ***RELB*** | *SERAC1* | *STAT2* | *TIRAP* | ***TRIM22*** | ***ZNF34*** |
| *MAVS* | ***NCKAP1L*** | ***PARP1*** | *PRKCD* | ***RELN*** | *SERPING1* | *STAT3* | *TLR2* | ***TRIM69*** | ***ZNF341*** |
| *MBL2* | *NCSTN* | ***PAX1*** | *PRKDC* | *RET* | ***SGPL1*** | *STAT4* | *TLR3* | *TRNT1* |  |
| *MC2R* | ***NFAT5*** | ***PAX5*** | *PRPS1* | ***RFWD3*** | ***SH2B3*** | *STAT5A* | *TLR4* | ***TSPAN14*** |  |
| ***MCM10*** | ***NFE2L2*** | *PBX1* | ***PSEN1*** | *RFX5* | *SH2D1A* | *STAT5B* | *TLR5* | *TTC37* |  |
| *MCM4* | *NFKB1* | *PCCA* | *PSENEN* | *RFXANK* | *SH3BP2* | ***STAT6*** | *TLR7* | *TTC7A* |  |
| ***MED13L*** | *NFKB2* | *PCCB* | ***PSMA3*** | *RFXAP* | *SH3BP5* | *STIM1* | *TLR8* | ***TTR*** |  |
| *MEFV* | *NFKBIA* | *PDCD1* | ***PSMB10*** | *RHOH* | ***SH3KBP1*** | *STING1* | *TLR9* | ***TUBGCP3*** |  |
| ***MICA*** | ***NFKBID*** | *PEPD* | ***PSMB4*** | *RIPK1* | *SKIV2L* | *STK4* | *TMC6* | *TYK2* |  |
| *MLPH* | *NFKBIL1* | *PGM3* | *PSMB8* | *RIPK3* | ***SLC13A4*** | ***STN1*** | *TMC8* | ***UBA1*** |  |
| ***MOGS*** | *NHEJ1* | *PHF11* | ***PSMB9*** | *RMRP* | *SLC22A4* | *STX11* | *TNF* | ***UBE2T*** |  |
| ***MPI*** | *NHP2* | *PIGA* | ***PSMG2*** | *RNASEH2A* | *SLC29A3* | *STXBP2* | ***TNFAIP3*** | *UNC119* |  |
| *MPO* | *NKX2-5* | *PIK3CD* | *PSTPIP1* | *RNASEH2B* | *SLC35A1* | *TANK* | *TNFRSF11A* | *UNC13D* |  |
| ***MR1*** | *NLRC3* | ***PIK3CG*** | *PTCRA* | *RNASEH2C* | *SLC35C1* | *TAP1* | *TNFRSF13B* | *UNC93B1* |  |
| *MRE11* | *NLRC4* | *PIK3R1* | *PTEN* | *RNF168* | *SLC37A4* | *TAP2* | *TNFRSF13C* | *UNG* |  |
| *MRTFA* | *NLRP1* | *PLA2G7* | ***PTPN2*** | *RNF31* | *SLC39A4* | *TAPBP* | *TNFRSF1A* | *UPB1* |  |
| *MS4A1* | *NLRP12* | *PLCG2* | *PTPN22* | ***RNU4ATAC*** | ***SLC39A7*** | *TAZ* | *TNFRSF4* | *USB1* |  |
| *MSH5* | *NLRP2* | ***PLEKHM1*** | *PTPRC* | ***RORC*** | *SLC46A1* | *TBK1* | ***TNFRSF9*** | ***USP18*** |  |
| ***MSH6*** | *NLRP3* | *PLG* | *PYCARD* | *RPSA* | ***SLC7A7*** | *TBX1* | ***TNFSF11*** | *VAV1* |  |
| ***MSN*** | *NLRP7* | *PMM2* | *RAB27A* | *RSPH9* | ***SLP76(LCP2)*** | ***TBX21*** | *TNFSF12* | *VPS13B* |  |
| *MST1* | *NOD2* | *PMS2* | *RAC2* | *RTEL1* | *SLX4* | *TCF3* | ***TNFSF13*** | *VPS45* |  |
| *MTHFD1* | *NOP10* | *PNP* | ***RAD51*** | ***SAMD3*** | *SMARCAL1* | *TCIRG1* | *TNFSF13B* | *WAS* |  |
| ***MTPAP*** | *NOS2* | ***POLA1*** | *RAD51C* | ***SAMD9*** | ***SMARCD2*** | *TCN2* | ***TNIP1*** | *WDR1* |  |
| *MVK* | *NRAS* | ***POLD1*** | *RAG1* | ***SAMD9L*** | ***SNORA31*** | *TERC* | ***TOM1*** | *WDR5* |  |
| ***MX1*** | *NSMCE3* | ***POLD2*** | *RAG2* | *SAMHD1* | ***SNX10*** | *TERT* | ***TOP2B*** | *WIPF1* |  |
| *MYD88* | ***OAS1*** | *POLE* | ***RANBP2*** | ***SART3*** | ***SOCS1*** | ***TET2*** | ***TP53*** | *WRAP53* |  |
| *MYO5A* | ***ODC1*** | ***POLE2*** | ***RASGRP1*** | *SBDS* | *SOCS4* | *TFRC* | ***TPP1*** | *XIAP* |  |
| *MYO5B* | *ORAI1* | ***POLR3A*** | *RASGRP2* | *SCGB1A1* | *SP110* | ***TGFB1*** | *TPP2* | ***XRCC2*** |  |
| ***MYSM1*** | ***OSMR*** | ***POLR3C*** | *RBCK1* | *SCGB3A2* | *SPINK5* | *TGFBR1* | *TRAC* | *XRCC6* |  |
| ***NBAS*** | *OSTM1* | ***POLR3E*** | ***RC3H1*** | *SCIMP* | ***SPPL2A*** | ***TGFBR2*** | *TRAF3* | *ZAP70* |  |

**Table 2: Presentation of Phenotypes in the Cohort (n=95 patients)**

| **ID^*^** | **Age (symptom debut)** | **Age (time for genetic analysis)** | **Gender (f/m)** | **Family history of PID** | | | **Recurrent/ opportunistic infections (including type of infectious agent when known)** | | **Immune dysregulation phenotype (autoimmunity, autoinflammation, lymphoproliferation, malignancy, allergy, others) and other clinical features (key words)** | **Genetic findings (gene name(s) and ACMG classification^†^** | **Clinical diagnosis (ESID 2019) (n=86) /Genetic diagnosis in patients with causal variants^‡^ (n=8)** | **Immunological findings** | **HPO codes** |
| --- | --- | --- | --- | --- | --- | --- | --- | --- | --- | --- | --- | --- | --- |
| **Unclassified Antibody Deficiency** | | | | | |  |  | |  |  |  |  |  |
| **1S** | 0-10 | 60-70 | f | _ | | | Recurrent upper respiratory tract infections. | | Fatigue and low-grade fever. | _ | Unclassified antibody deficiency | IgA↓ and episodes of IgG1 and IgG3↓ | Fatigue HP:0012378, Low-grade fever HP:0011134, Decreased circulating antibody level HP:0004313 |
| **2S** | 40-50 | 40-50 | f | 1^st^ degree relative with AI | | | Recurrent oral candidiasis. VZV. | | AI. Recurrent abdominal pain. | _ | Unclassified antibody deficiency | IgM↓. Decreased specific antibody response to vaccination (pneumococcus)↓ | Gastrointestinal obstruction HP:0004796, Autoimmunity HP:0002960, Decreased circulating antibody level HP:0004313, Thyroid adenoma HP:0000854, Lymphadenitis HP:0002840, Complete or near-complete absence of specific antibody response to unconjugated pneumococcus vaccine HP:0410300 |
| **3T** | 0-10 | 30-40 | f | 1^st^ degree relative with unusual/severe or recur-rent infection | | | Otitis media in childhood. Recurrent respiratory tract infections and sinusitis. Recurrent herpes. | | Asthma. Allergy. HPV induced dysplasia. | _ | Unclassified antibody deficiency | IgM and IgG3↓. IgA↑ | Asthma HP:0002099, Decreased circulating antibody level HP:0004313, Hypertension HP:0000822, Cervical dysplasia HP:0032131, Recurrent oral herpes HP:0410028, Recurrent genital herpes HP:0032157, Allergy: HP:0012393, Recurrent bacterial infections HP:0002718 |
| **4T** | 0-10 | 30-40 | f | _ | | | Congenital toxoplasmosis. Recurrent upper respiratory tract infections (sinusitis, otitis) and pneumonia. Invasive salmonellosis. | | AI. Asthma. Allergy. Arthritis. | 2 VUSs in *NCF2* | Unclassified antibody deficiency | B-cell count and IgG3↓. Reduced response to vaccination. | Decreased specific antibody response to protein vaccine HP:0410294, Autoimmunity HP:0002960, Recurrent bacterial infections HP:0002718, B lymphocytopenia HP:0010976, Decreased circulating antibody level HP:0004313, Asthma HP:0002099, Allergy HP:0012393, Arthritis HP:0001369 |
| **5S** | 0-10 | 50-60 | m | _ | | | Recurrent pulmonary infections (bacterial) | | Allergy. Atopic dermatitis. Severe asthma with reduced lung function and bronchiectasis. Uncertain whether B cell deficiency is caused by prolonged steroid treatment. | 2 VUSs and 1 LP in *IL17RA* | Unclassified antibody deficiency | B-cells↓. IgG1, IgG2, IgA and IgM↓. Reduced response to pneumococcus vaccination. | Allergy: HP:0012393, Atopic dermatitis HP:0001047, Asthma HP:0002099, Reduced FEV1/FVC ratio HP:0030877, Decreased circulating antibody level HP:0004313, Complete or near-complete absence of specific antibody response to unconjugated pneumococcus vaccine HP:0410300 |
| **6S** | 20-30 | 20-30 | f | _ | | | _ | | Unexplained fever in pregnancy. Hepato-splenomegaly. Lymphadenitis. Granulomatous inflammation in lymph node. | _ | Unclassified antibody deficiency | B-cells↓. IgG3↓. | Maternal fever in pregnancy HP:0030244, Hepatosplenomegaly HP:0001433, Lymphadenitis HP:0002840, Decreased circulating antibody level HP:0004313 |
| **7S** | 50-60 | 60-70 | f | _ | | | Recurrent pulmonary infections and sinusitis (bacterial) (respond to AB, no positive cultures) | | AI | VUS in *GSN^§^* | Unclassified antibody deficiency | IgG2, IgG3 and IgM↓. | Asthma HP:0002099, Decreased circulating antibody level HP:0004313, Autoimmunity HP:0002960 |
| **8S** | 50-60 | 60-70 | f | Relatives with unusual/severe or recurrent infection | | | Recurrent abscess formation after extraction of teeth. Recurrent upper respiratory tract infections. | | _ | _ | Unclassified antibody deficiency | IgG↓ | Decreased specific antibody response to vaccination HP:0032140, Decreased circulating antibody level HP:0004313 |
| **9S** | 0-10 | 40-50 | f | _ | | | Recurrent otitis media in childhood. Pneumonia (bacterial). Chronic sinusitis (Haemophilus influenzae). | | Bronchiectasis. Recurrent basal cell carcinoma. | VUS in *TNFRSF13B,* | Unclassified antibody deficiency | IgA↓, IgG2↓ and IgG3↓. Somatic hypermutation↓ | Basal cell carcinoma HP:0002671, Decreased circulating antibody level HP:0004313, Recurrent respiratory infections HP:0002205, Bronchiectasis HP:0002110 |
| **10FP** | 40-50 | 40-50 | f | 1^st^ degree relative with AI | | | Milder infections primarily of the upper respiratory tract. | | AI. Arterial thrombosis. |  | Unclassified antibody deficiency | IgG↓ | Ulcerative colitis HP:0100279, Decreased circulating antibody level HP:0004313, Rheumatoid arthritis HP:0001370, Hashimoto thyroiditis HP:0000872, Arterial thrombosis HP:0004420 |
| **10FC** | 0-10 | 10-20 | m | 1^st^ degree relative with AI | | | _ | | AI. Allergy. Bronchitis. Delayed puberty. | _ | Suspicion of unclassified disorders of immune dysregulation | N/A | Rheumatoid arthritis HP:0001370, Recurrent bronchitis HP0002837, Rhinitis HP:0012384, Delayed puberty HP:0000823 |
| **11S** | 0-10 | 30-40 | f | _ | | | Recurrent upper respiratory infections, pneumonia and in childhood otitis media. | | Growth delay. Early menopause. Osteoporosis. | LP in *RNF168^‡^* | Unclassified antibody deficiency | B cells↓, IgA ↓ | Osteoporosis HP:0000939, Decreased circulating antibody level HP:0004313, Premature ovarian insufficiency HP:0008209, Growth delay HP:0001510 |
| **12S** | 20-30 | 70-80 | f | _ | | | Recurrent bacterial meningitis (pneumococcus) and upper respiratory tract infections | | _ | _ | Unclassified antibody deficiency/ Suspicion of specific antibody deficiency (SPAD) | IgM↓. Reduced response to polysaccharide vaccination. | Decreased specific antibody response to protein vaccine HP:0410294, Meningitis HP:0001287, |
| **13S** | 60-70 | 70-80 | f | _ | | | Recurrent pneumonia (6-12 per year) without need for hospitalization. | | COPD. Recurrent diarrhea. | _ | Unclassified antibody deficiency/ Suspicion of HIGM syndrome | IgG↓. IgM↑ | Chronic pulmonary obstruction HP:0006510, Diarrhea HP:0002014, Recurrent pneumonia HP:0006532, Decreased circulating antibody level HP:0004313 |
| **14FP** | 10-20 | 50-60 | f | 1^st^ degree relatives with PID | | | Recurrent upper respiratory tract infections, pneumonia and sinusitis. | | Allergy. | VUS in *NLRP2* | _ | Normal | Bowel diverticulosis HP:0005222, Recurrent respiratory infections HP:0002205, Allergy: HP:0012393 |
| **14FC1** | 0-10 | 10-20 | f | 1^st^ degree relatives with PID | | | Recurrent upper respiratory tract infections, pneumonia and sinusitis. | | Allergy. UNS gastrointestinal symptoms. | VUS in *NLRP2* | Unclassified antibody deficiency | IgG↓ | Recurrent respiratory infections HP:0002205, Allergy: HP:0012393 |
| **14FC2** | 0-10 | 10-20 | m | 1^st^ degree relatives with PID | | | Recurrent upper respiratory tract infections and sinusitis. | | Allergy. | VUS in *NLRP2* | Unclassified immunodeficiencies | CD4+ T-cell concentration↓ | Recurrent respiratory infections HP:0002205, Allergy: HP:0012393 |
| **14FC3** | 0-10 | 10-20 | m | 1^st^ degree relatives with PID | | | Recurrent upper respiratory tract infections, pneumonia and sinusitis. | | Allergy. UNS gastrointestinal symptoms. | VUS in *NLRP2* | Unclassified antibody deficiency | IgA↓ IgG↓ SHM↓ | Recurrent respiratory infections HP:0002205, Allergy: HP:0012393 |
| **15FP^2^** | 0-10 | 40-50 | f | 1^st^ degree relatives with PID | | | Severe VZV infection in childhood. | | Proliferative ischemic retinopathy. Hyper viscosity syndrome. | VUS in *NLRP3* | Unclassified antibody deficiency | B cells↓ and IgM↓. Vaccination response↓. | Hypertension HP:0000822, Decreased circulating antibody level HP:0004313, Decreased specific antibody response to protein vaccine HP:0410294, Decreased circulating antibody level HP:0004313 |
| **15FC1^2^** | 0-10 | 10-20 | f | 1^st^ degree relatives with PID | | | Recurrent upper respiratory tract infections and pneumonia. | | Asthma | VUS in *NLRP3* | Unclassified antibody deficiency | IgG↓, IgG1↓ and IgM↓ | Recurrent respiratory infections HP:0002205, Asthma HP:0002099 |
| **15FC2**^¶^ | 0-10 | 10-20 | m | 1^st^ degree relatives with PID | | | Recurrent upper respiratory tract infections and pneumonia. | | _ | VUS in *NLRP3* | CVID | IgA↓, IgG, IgG1↓ and IgM↓. CD4+ T-cell concentration↓ Somatic hypermutation↓. Vaccination response↓ | Recurrent respiratory infections HP:0002205 |
| **Selective IgM Deficiency** | | | |  |  | |  | |  |  |  |  |  |
| **16S** | 0-10 | 40-50 | f | Relatives with similar phenotype | | | _ | | Autoinflammation. Recurrent oral ulcers/stomatitis. Diarrhea. Myalgia. Arthralgia. Fatigue. Benign medullar ependymoma. | VUS in *TNFRSF13C,* | Selective IgM deficiency / Suspicion of unclassified autoinflammatory diseases | IgM↓ | Ependymoma HP:0002888, Recurrent aphthous stomatitis HP:0011107, Decreased circulating antibody level HP:0004313 |
| **IgG-Subclass Deficiency** | | | |  |  | |  | |  |  |  |  |  |
| **17S** | 10-20 | 50-60 | f | 1^st^ degree relative with asthma and allergy | | | Recurrent abscess formation. Recurrent mucocutaneous candidiasis. Recurrent pneumonia | | Asthma and secondary adrenal insufficiency | VUS in *DOCK8* | Isolated IgG subclass deficiency | IgG and IgG3↓. Reduced response to pneumococcus vaccination. | Recurrent abscess formation HP:0002722,  Recurrent vulvovaginal candidiasis HP:0012204, asthma: HP:0002099, Migraine HP:0002076, Hypertension HP:0000822,  Secondary adrenal insufficiency HP:0011734, Decreased circulating antibody level HP:0004313, Tremor HP:0001337, Decreased specific antibody response to protein vaccine HP:0410294 |
| **18S** | 0-10 | 20-30 | f | _ | | | Recurrent pneumonia, otitis media, sinusitis and tonsilitis (bacterial). | | Recurrent fever | _ | Isolated IgG subclass deficiency | Low IgG3↓. Somatic hypermutation↓. | Schwannoma HP:0100008, Recurrent fever HP:0001954, Decreased circulating IgG3 level HP:0032137, Recurrent upper respiratory tract infections HP:0002788, Decreased circulating antibody level HP:0004313 |
| **19S** | 10-20 | 50-60 | f | _ | | | Recurrent pneumonia and sinusitis (bacterial) | | Asthma. Allergy. Bronchiectasis. Chronic inflammation of the jaw. Cholecystectomy. Appendectomy. | VUS in *FLG* | Isolated IgG subclass deficiency | IgG3↓ | Allergy: HP:0012393, Asthma HP:0002099, Bronchiectasis HP:0002110. Decreased circulating antibody level HP:0004313 |
| **20S** | 0-10 | 10-20 | f | _ | | | Recurrent otitis media and tonsillitis in childhood. Skin abscesses, perioral dermatitis and impetigo. Recurrent genital herpes infections and one VZV episode. | | Allergy. Tonsillectomy. | _ | Isolated IgG subclass deficiency | IgG3↓ | Recurrent genital herpes HP:0032157, Allergy: HP:0012393, Cutaneous abscess HP:0031292. Decreased circulating antibody level HP:0004313 |
| **21S** | 50-60 | 50-60 | f | Relatives with AI | | | _ | | Sterile abscess of the spleen. MGUS. Hypertension. | _ | Isolated IgG subclass deficiency | B cell count ↓. IgG2 and IgG3↓ | Asthma: HP:0002099, Decreased circulating antibody level HP:0004313, Paraproteinemia HP:0031047, Splenic abscess HP:0025059, Bowel diverticulosis HP:0005222, B lymphocytopenia HP:0010976 |
| **22S** | 20-30 | 50-60 | m | Relatives with AI | | | Recurrent pneumonia. Septic arthritis. | | AI. Asthma. Allergy. Diarrhea. Fatigue. | VUS in *CARD11* | Isolated IgG subclass deficiency | IgG3↓ | Hypothyroidism HP:0000821, Allergy HP:0012393, Asthma: HP:0002099, Gastrointestinal eosinophilia HP:0032064, Decreased circulating antibody level HP:0004313, Septic arthritis HP:0003095 |
| **23S** | 30-40 | 40-50 | f | _ | | | Recurrent upper respiratory infections and pneumonia (bacterial). Abscess of the skin. | | Asthma. | VUS in *IKZF1,* P in *BRCA2^§^* | Isolated IgG subclass deficiency | IgG2↓ | Decreased circulating antibody level HP:0004313, Asthma HP:0002099, Colitis HP:0002583, Cutaneous abscess HP:0031292, Recurrent urinary tract infections HP:0000010 |
| **24S** | 40-50 | 70-80 | m | _ | | | Recurrent pneumonia (bacterial). | | Recurrent fever without elevated CRP. | VUS in *MEFV,* VUS in *IGLL1* | Isolated IgG subclass deficiency / Suspicion of Unclassified autoinflammatory  diseases | IgG3↓. CD8 T-cells↓ | Recurrent fever HP:0001954, Decreased circulating antibody level HP:0004313, Lymphopenia HP:0001888, Bronchiectasis HP:0002110, Complete or near-complete absence of specific antibody response to unconjugated pneumococcus vaccine HP:0410300 |
| **Complement Component 2 Deficiency** | | | | | | |  | |  |  |  |  |  |
| **25S** | 0-10 | 30-40 | f | 1^st^ degree relative with unusual/severe or recurrent infection | | | Recurrent pneumonia and tonsillitis. Severe myelitis and meningitis (bacterial). | | Pulmonary emphysema. Allergy. Atopic dermatitis. Asthma. | LP in *C2^‡^* | Complement component 2 deficiency | Absent activation of the classical complement pathway. | Allergy: HP:0012393, Asthma: HP:0002099, Atopic dermatitis HP:0001047, venous thrombosis: HP:0004936, Tetraplegia HP:0002445, Myelitis HP:0012486, Emphysema HP:0002097, Reduced total lung capacity HP:0033169 |
| **T-cell Deficiency and Combined Immunodeficiency** | | | | | | | |  |  |  |  |  |  |
| **26T** | 0-10 | 20-30 | f | _ | | | Recurrent pneumonia. Viral meningitis. Persistent HPV infection and dysplasia. | |  | _ | Severe combined immunodeficiency (SCID) | CD4 T-cells and RTE↓. B-cells↓. | Meningitis HP:0001287, Persistent human papillomavirus infection HP:0020114, Recurrent pneumonia HP:0006532, Severe combined immunodeficiency HP:0004430, Recurrent herpes HP:0005353 |
| **27S** | 50-60 | 70-80 | f | _ | | | Recurrent abscess formation in skin and in the mouth (Staphylococcus). | | Chronic inflammation, abscess and fistula of the skin after injections or teeth extraction. Abdominal cysts. Hepato-splenomegaly. Elevated liver enzymes. |  | Combined immunodeficiency (CID) | CD4 and CD8 T cell counts↓. RTE↓ | Vitamin B12 deficiency HP:0100502, Recurrent abscess formation HP:0002722, Combined immunodeficiency HP:0005387 |
| **28S** | 20-30 | 20-30 | m | _ | | | Severe molluscum contagiosum infection (pox virus) | | Allergy. Eczema. Pure red cell anaemia. Cardiomyopathy. Renal insufficiency. | _ | Combined immunodeficiency (CID) | CD4 T cell↓ naive CD4 T-cells↓ g/d T-cells↑ | Combined immunodeficiency HP:0005387, Allergy: HP:0012393, Pure red cell aplasia HP:0012410, Renal insufficiency HP:0000083, Cardiomyopathy HP:0001638, Molluscum contagiosum HP:0032163 |
| **29S** | 0-10 | 20-30 | f | 1^st^ degree relative with AI. 2^nd^ degree relative with recurrent pneumonia, IgA↓ and splenomegaly. | | | Recurrent pneumonia and otitis media in childhood. Severe VZV infection. Abscess of the skin. GI infection. Appendicitis. Meningitis (unknown agent ). | | Atopic dermatitis. Reduced lung function and bronchiectasis. | VUS in *STAT3,* VUS in *OAS1* | Combined immunodeficiency (CID) | B-cell count↓ and T-cell count↓. Somatic hypermutation↓ | Recurrent pneumonia HP:0006532, Atopic dermatitis HP:0001047, Bronchiectasis HP:0002110, Reduced FEV1/FVC ratio HP:0030877, Joint hypermobility HP:0001382, Combined immunodeficiency HP:0005387 |
| **30T** | 0-10 | 20-30 | m | Consanguinity, parents are cousins | | | Respiratory infections. Mollusculum contagiosum and recurrent VZV. | | Squamous cell carcinoma. AI. | _ | Combined immunodeficiency (CID) / Suspicion of Nijmegen breakage syndrome | Severe T-cell deficiency. B-cells ↓ | Hashimoto thyroiditis HP:0000872, Osteoporosis HP:0000939, Molluscum contagiosum HP:0032163, Azoospermia HP:0000027, Ptosis HP:0000508, HP:0002860, Squamous cell carcinoma, Kyphosis HP:0002808, Facial palsy HP:0010628, Combined immunodeficiency HP:0005387 |
| **31S** | 50-60 | 50-60 | f | _ | | | _ | | COPD. | VUS in *TNFAIP3* | Unclassified immunodeficiencies / Suspicion of HIGM syndrome | IgG↓ and IgA↓, IgM↑. Somatic hypermutation↓. Isotype switched memory B-cells: reduced fraction with normal concentration. | Chronic lung disease HP:0006528, Fatigue HP:0012378 |
| **32S** | 0-10 | 30-40 | m | 1^st^ degree relatives with atopic dermatitis | | | Recurrent cutaneous infections (bacterial). | | Allergy. Asthma. Severe atopic dermatitis. Keratoconus. |  | Unclassified immunodeficiencies/ Suspicion of HIES | N/A, IgE↑ | Keratoconus HP:0000563, Allergy: HP:0012393, Asthma: HP:0002099, Atopic dermatitis HP:0001047, |
| **Pathogen-Specific Immunodeficiency** | | | | | | |  | |  |  |  |  |  |
| **33S** | 10-20 | 20-30 | m | 1^st^ degree relative with AI | | | Severe manifestations of viral infections (EBV and CMV). | | Severe EBV infection complicated by splenic rupture and hepatitis. Severe CMV infection. |  | Unclassified immunodeficiencies/ Suspicion of XLP | Inverted CD4/CD8 T-cell ratio and immune activation. | Severe Epstein Barr virus infection HP:0031693, Splenic rupture HP:0012223, Lymphocytosis HP:0100827 |
| **34S** | 60-70 | 70-80 | m | _ | | | Recurrent pneumonia. VZV meningitis. | | Clinical suspicion of bare lymphocyte syndrome. Allergy. Asthma. Atopic dermatitis. Renal failure. | _ | Unclassified immunodeficiency es | CD8 T-cell concentration ↓. B-cell count ↓ | Focal segmental glomerulosclerosis HP:0000097, Severe varicella zoster infection HP:0032170, Recurrent pneumonia HP:0006532, Asthma: HP:0002099, Atopic dermatitis HP:0001047, Bronchiectasis HP:0002110, Hypertension HP:0000822, Allergy: HP:0012393, T lymphocytopenia HP:0005403, B lymphocytopenia HP:0010976 |
| **35T** | 20-30 | 20-30 | m | 1^st^ degree relatives with recurrent HSV infections | | | HSV encephalitis | | _ | _ | _ | Normal | Herpes simplex encephalitis HP:0012302 |
| **36S** | 50-60 | 50-60 | m | _ | | | Recurrent VZV encephalomyelitis with neurologic complications. | | _ | VUS in *TLR9,* VUS in *IL17F* | _ | N/A | Severe varicella zoster infection HP:0032170 |
| **37S** | 20-30 | 60-70 | f | 1^st^ - and 2^nd^ degree relatives with PID | | | Recurrent herpes zoster (*5). Upper respiratory tract infections and sinusitis (Aspergillus). | | AI. Secondary adrenal insufficiency. | LP in *C2* | Unclassified immunodeficiencies | Reduced activation of the classical complement pathway. IgM↑. Absent antibody response to pneumococcus vaccine. MBL deficiency (XA/XA). | Osteopenia HP:0000938,  Secondary adrenal insufficiency HP:0011734, Rheumatoid arthritis HP:0001370, Complete or near-complete absence of specific antibody response to unconjugated pneumococcus vaccine HP:0410300 |
| **38S** | 20-30 | 30-40 | m | _ | | | Chronic pulmonary aspergillosis | | _ | _ | _ | Normal | Recurrent Aspergillus infections HP:0002724 |
| **39FA1** | 60-70 | 60-70 | f | Two 1^st^ degree relatives with unusual/severe or recurrent infection | | | Bacterial meningitis (pneumococcus) | | _ | VUS in *TLR3* | _ | Normal (including complement function and antibody response, TLR function not investigated) | Meningitis HP:0001287 |
| **39FA2** | 60-70 | 70-80 | f | Two 1^st^ degree relatives with unusual/severe or recurrent infection | | | Bacterial meningitis (pneumococcus) | | _ | VUS in *TLR3* | _ | N/A | Meningitis HP:0001287 |
| **40S** | 0-10 | 20-30 | f | _ | | | Persistent HPV infection causing papillomas in the respiratory tract, HPV type 6 and 11. | | B12 deficiency. | VUS in *TINF2* | _ | Normal | Laryngeal papilloma HP:0033001, Vitamin B12 deficiency HP:0100502 |
| **Disorders of Immune Regulation** | | | | | |  |  | |  |  |  |  |  |
| **41S** | 10-20 | 10-20 | f | 1^st^ degree relative with AI | | | Recurrent sinusitis, upper respiratory tract infections and pneumonia (bacterial). Acne and skin infections (Staphylococcus aureus). | | Clinical suspicion of Systemic autoinflammatory disorder. Recurrent fever. Fatigue. Gastric micronodular neuroendocrine tumours. | _ | Unclassified disorders of immune dysregulation/ Suspicion of Unclassified autoinflammatory diseases | IgM↑ and IgG1↑ | Recurrent respiratory infections HP:0002205, Recurrent urinary tract infections HP:0000010, Hashimoto: HP:0000872, Uveitis HP:0000554, Recurrent fever HP:0001954, Fatigue HP:0012378 |
| **42T** | 0-10 | 20-30 | f | 1^st^ degree relative with unusual/severe or recurrent infection | | | Recurrent otitis and pneumonia (bacterial) | | ITP. Splenectomy. Recurrent fever. UNS gastro intestinal symptoms. Dermal rash. | _ | Unclassified disorders of immune dysregulation | IgG↑ | ITP: HP:0001973, Recurrent bacterial infections HP:0002718, Asthma HP:0002099, Contact dermatitis HP:0032282, Narcolepsy HP:0030050, cholecystectomy, tonsillectomy and appendectomy (no HPOs). Recurrent fever HP:0001954, Butterfly rash: HP:0025300, Bloody diarrhea HP:0025085, Arthralgia HP:0002829, Abnormal oral mucosa morphology HP:0011830, fibromyalgia (no HPO), Cervical dysplasia HP:0032131. |
| **43T** | 0-10 | 30-40 | f | 1^st^ degree relatives with AI, asthma and allergy. | | | Recurrent tonsillitis and pneumonia(bacterial) | | AI. Asthma. Diarrhea | _ | Unclassified disorders of immune dysregulation | CD4+ and CD8+ T-cells↑ | Asthma: HP:0002099, Diarrhea HP:0002014, Polycystic ovaries HP:0000147. Autoimmunity HP:0002960 (T1DM: HP:0100651, Celiac disease HP:0002608), Allergy: HP:0012393, Recurrent bacterial infections HP:0002718 |
| **Familial Chronic Mucocutanous Candidiasis** | | | | | | | |  |  |  |  |  |  |
| **44T** | 0-10 | 20-30 | m | _ | | | Recurrent pneumonia, skin infections and abscess formation. Chronic mucocutaneous candidiasis. Recurrent HSV infections. | | AI. Lymphopenia | P in *STAT1^‡^* | Familial chronic mucocutaneous candidiasis | CD4 and CD8 T cell counts↓. RTE↓. B-cells↓ | Chronic mucocutaneous candidiasis HP:0002728, Bronchiectasis HP:0002110, Recurrent bacterial infections HP:0002718, Autoimmunity HP:0002960, Combined immunodeficiency HP:0005387, Recurrent abscess formation HP:0002722, Decreased proportion of CD4-positive T cells HP:0032218, B lymphocytopenia HP:0010976 |
| **45S** | 0-10 | 50-60 | f | 1^st^ degree relatives with similar phenotype | | | Chronic mucocutaneous candidiasis.  Recurrent mouth infections, pneumonia and sinusitis (bacterial). Recurrent HSV infections. | | Alopecia. Squamous cell carcinoma. Cervical dysplasia. | P in *STAT1^‡^* | Familial chronic mucocutaneous candidiasis | CD4 T cells↓. T cell proliferation↓ | Chronic mucocutaneous candidiasis HP:0002728, Alopecia HP:0001596, Nail dystrophy HP:0008404, Cervical dysplasia HP:0032131 |
| **46S** | 0-10 | 30-40 | f | _ | | | Recurrent abscess formation in skin. Oral and oesophageal candidiasis. Sinusitis. Mouth and jaw infections. | | Prolactinoma. Oral ulcers | P in *STAT1^‡^* | Familial chronic mucocutaneous candidiasis | CD4+ T-cells↓ IgG↓ (total and IgG2) | Prolactinoma HP:0040278, Recurrent abscess formation HP:0002722, Decreased proportion of CD4-positive T cells HP:0032218, Decreased circulating antibody level HP:0004313, Oral ulcer HP:0000155 |
| **Autoimmune Lymphoproliferative Syndromes** | | | | | | | |  |  |  |  |  |  |
| **47T** | 0-10 | 30-40 | f | _ | | | Recurrent respiratory tract infections and meningitis. Recurrent HSV and HPV. JC virus encephalitis. | | In childhood Autoimmune Lymphoproliferative Syndrome. Detection of a somatic FAS variant. In adulthood development of JC virus induced Progressive Multifocal Leukoencephalopathy. | VUS in *RAG1* | ALPS type 1A/ Unclassified immunodeficiency es | Low concentration of memory B-cells, CD4+ T-cells low in the normal range. (60% DNT in adolescence). | Recurrent respiratory infections HP:0002205, Meningitis HP:0001287, Persistent human papillomavirus infection HP:0020114, |
| **48T** | 0-10 | 30-40 | m | _ | | | Recurrent respiratory and gastrointestinal infections. | | AI. | P in *CTLA4^‡^* | Autoimmune lymphoproliferative syndrome, type V | Severe T-cell deficiency. IgA and IgG ↓. Somatic hypermutation↓. | Autoimmunity HP:0002960, Combined immunodeficiency HP:0005387, Recurrent bacterial infections HP:0002718 |
| **Autoinflammatory Disorders and Periodic Fever Syndromes** | | | | | | | | |  |  |  |  |  |
| **49S** | 10-20 | 20-30 | m | _ | | | _ | | Recurrent fever and arthralgia. UNS gastrointestinal symptoms and CRP↑. | VUS in *NFKB1* | Unclassified autoinflammatory diseases | Normal | Recurrent fever HP:0001954, Arthralgia HP:0002829 |
| **50T** | 0-10 | 10-20 | f | _ | | | _ | | Recurrent fever with lower respiratory tract symptoms and CRP↑. | _ | Unclassified autoinflammatory diseases | IgD ↑ | Recurrent fever HP:0001954 |
| **51FA1** | 10-20 | 20-30 | f | _ | | | _ | | Recurrent fever and UNS gastrointestinal symptoms. Clinical suspicion of Hyper IgD Syndrome. | _ | Unclassified immunodeficiency es / Suspicion of unclassified autoinflammatory diseases | N/A, IgD ↑ | Recurrent fever HP:0001954, Asthma HP:0002099, Increased circulating IgD level HP:0410246 |
| **51A2** | 20-30 | 20-30 | f | _ | | | _ | | Recurrent fever and gastrointestinal symptoms | _ | Suspicion of Unclassified autoinflammatory diseases | N/A | Recurrent fever HP:0001954 |
| **52S** | 0-10 | 10-20 | f | 1^st^ degree relatives with similar phenotype | | | Recurrent conjunctivitis and otitis media in childhood. | | Recurrent fever with fatigue, headache and arthralgia. | VUS in *NLRP3* | Muckle-Wells Syndrome | Normal | No Muckle-Wells HPO. |
| **53S** | 0-10 | 20-30 | m | _ | | | _ | | Recurrent fever with oral ulcers. | P in *TCIRG1,* VUS in *MEFV,* VUS in *PLCG2* | Suspicion of Unclassified autoinflammatory diseases | Normal | Recurrent fever HP:0001954, Oral ulcer HP:0000155 |
| **54S** | 20-30 | 30-40 | f | _ | | | _ | | Allergy. Atopic dermatitis. Asthma in childhood. Recurrent fever. | P in *FLG* | Suspicion of unclassified autoinflammatory diseases | IgE↑ | Asthma: HP:0002099, Neutrophilia HP:0011897, Eosinophilia HP:0001880, Increased circulating IgE level HP:0003212 |
| **55S** | 20-30 | 20-30 | f | 1^st^ degree relatives with AI | | | Recurrent aphthous stomatitis and verrucae of the skin. VZV infection. | | Recurrent fever with mouth-, skin and genital vesicles. Effect of prednisolone. | _ | Suspicion of unclassified autoinflammatory diseases | Normal | Recurrent fever HP:0001954, Verrucae HP:0200043, Recurrent aphthous stomatitis HP:0011107 |
| **56FP** | 40-50 | 40-50 | f | 1^st^ degree relative with AI | | | Recurrent infections of the skin (bacterial). | | Clinical suspicion of Systemic autoinflammatory disorder. Recurrent fever with mouth ulcers. Hydrocephalus. | _ | Suspicion of Unclassified autoinflammatory diseases | Normal | Recurrent fever HP:0001954, Solar urticaria HP:0410137, Oligoarthritis HP:0040313, Cutis marmorata HP:0000965, Patent foramen ovale HP:0001655, Hydrocephalus HP:0000238, Endometriosis HP:0030127, Oral ulcer HP:0000155 |
| **56FC** | 0-10 | 10-20 | m | 1^st^ degree relative with AI | | | _ | | AI. Malar rash. Mouth ulcers. Nephritis. | _ | Unclassified immunodeficiencies / Suspicion of Unclassified autoinflammatory diseases | Somatic hypermutation↓. Recurrent T, B and NK cell lymphopenia. | Systemic lupus erythematosus HP:0002725, Skin rash 0000988, Oral ulcer HP:0000155 / Nephritis HP:0000123 / lymphopenia HP:0001888 |
| **57S** | 10-20 | 20-30 | m | _ | | | Recurrent tonsillitis. Rectal sphincter abscess. | | Recurrent fever with sore throat, cough and abdominal pain. | _ | Suspicion of unclassified autoinflammatory diseases | N/A | Recurrent fever HP:0001954, Anorectal abscess HP:0033150, (Lactose intolerance HP:0004789) |
| **58T** | 0-10 | 20-30 | m | _ | | | _ | | Recurrent fever with effect of Colchicine treatment. Recurrent arthritis. | _ | Suspicion of unclassified autoinflammatory diseases | N/A | Recurrent fever HP:0001954, Arthritis HP:0001369 |
| **59S** | 10-20 | 20-30 | f | _ | | | Recurrent tonsillitis. | | Recurrent fever with lymphadenitis, oedema of the larynx, arthralgia and erythema nodosum. Necrotic granuloma in lymph node. Effect of prednisolone. | VUS in *SERPING1* | Suspicion of unclassified autoinflammatory diseases | N/A | Lymphadenitis HP:0002840, Granuloma HP:0032252 |
| **60S** | 0-10 | 10-20 | m | 1^st^ degree relative with unusual/severe or recurrent infection | | | Recurrent tonsillitis. | | Recurrent fever every 2 weeks. Tonsillectomy. Cholesteatoma. Adenitis | _ | Suspicion of unclassified autoinflammatory diseases | N/A | Recurrent fever HP:0001954, Cholesteatoma HP:0009797 |
| **61T** | 10-20 | 20-30 | f | 1^st^ degree relative with AI | | | _ | | Recurrent mouth- and genital mucosa vesicles, arthralgia and fatigue. Effect of prednisolone treatment. | VUS in *PLCG2* | Suspicion of unclassified autoinflammatory diseases | N/A | Fatigue HP:0012378, Arthralgia HP:0002829 |
| **Defects of NFκ-B Signaling** | | | |  | | |  | |  |  |  |  |  |
| **62FP^#^** | 10-20 | 10-20 | f | 1^st^ degree relative with unusual/severe or recurrent infection | | | Severe generalized HSV-1 infection postpartum with hepatitis and causing renal failure. | | Hemophagocytic lymph histiocytosis. | LP in *CASP8,* VUS in *IKBKB,* 2 VUSs in *IRAK1* | Unclassified immunodeficiency es / Suspicion of defect of TLR/ NFκ-B signalling | Reduced TLR1/2 and 4 function. Reduced IFN response. | _ |
| **62FC^#^** | 0-10 | 0-10 | m | 1^st^ degree relative with unusual/severe or recurrent infection | | | Fatal neonatal herpes infection. | | Clinical suspicion of Hemophagocytic lymph histiocytosis. | LP in *CASP8,* VUS in *IKBKB,* VUS in *IRAK1* | Unclassified immunodeficiency es / Suspicion of defect of TLR/ NFκ-B signalling | _ | _ |
| **63S** | 40-50 | 40-50 | m | _ | | | _ | | Fever with CRP↑ | P in *NFKB1^‡^* | NFKB1 deletion/CVID12 | Isotype-switched memory B-cells concentration↓. IgM and IgG3↓. | Recurrent fever HP:0001954 |
| **Thymoma with Immunodeficiency** | | | | | | |  | |  |  |  |  |  |
| **64S** | 50-60 | 60-70 | m | _ | | | Recurrent sinusitis (bacterial). | | Goods syndrome. Basal cell carcinoma. Colitis. Retinal angiopathy. | VUS in *NFKB2* | Thymoma with immunodeficiency | IgG↓. CD4 T-cell count ↓ | Decreased circulating antibody level HP:0004313, Thymoma HP:0100522, Colitis HP:0002583, Basal cell carcinoma HP:0002671, Chronic diarrhea HP:0002028 |
| **65S** | 60-70 | 70-80 | f | Relatives with AI | | | Chronic oral candidiasis. Recurrent pneumonia (bacterial). | | Goods syndrome. AI. Bronchiectasis. Oesophageal stricture. | _ | Thymoma with immunodeficiency | B-cell count↓, IgA and IgG↓ Absent isotype-switched memory B cells. Somatic hypermutation↓ Reduced response to vaccination. T-cell proliferation↓, Inverted CD4/CD8 T-cell ratio. | Abnormality of the skin HP:0000951, Vitiligo HP:0001045, Thymoma HP:0100522, Bronchiectasis HP:0002110, Oesophageal stricture HP:0002043. Decreased circulating antibody level HP:0004313 |
| **Unclassified Immunodeficiency** | | | | | |  |  | |  |  |  |  |  |
| **66T** | 0-10 | 20-30 | f | _ | | | Recurrent pneumonia. | | Bronchiectasis. AI. Inflammation of the jaw. | _ | Unclassified immunodeficiency es | IgM and IgG ↑ (all subclasses) | Nasal polyposis HP:0100582, Autoimmunity HP:0002960, Increased circulating antibody level HP:0010702, Astigmatism HP:0000483, Bronchiectasis HP:0002110, Recurrent bacterial infections HP:0002718 |
| **67T** | 40-50 | 40-50 | f | _ | | | Recurrent upper respiratory tract infections and pneumonia (bacterial). | | Asthma. | _ | Unclassified immunodeficiency es | B-cells ↓ | B lymphocytopenia HP:0010976, Recurrent bacterial infections HP:0002718, Asthma HP:0002099 |
| **68S** | 0-10 | 40-50 | f | _ | | | Recurrent upper respiratory infections, pneumonia, tonsillitis, sinusitis and otitis media. | | Recurrent infections. Clinical suspicion of SLE. | _ | Unclassified immunodeficiency es | Proportion of isotype-switched memory B-cells↓ | Vasculitis HP:0002633, Arthralgia HP:0002829, Glomerulonephritis HP:0000099, Type II diabetes mellitus HP:0005978, Decreased circulating antibody level HP:0004313 |
| **69S** | 0-10 | 50-60 | f | _ | | | Recurrent upper respiratory tract infections and sinusitis (bacterial). Recurrent skin infections and abscess formation. | | Allergy. Asthma. Severe eczema in childhood. Clinical suspicion of Hyper IgE syndrome. | _ | Unclassified immunodeficiency es | (IgE↑ ) | Increased circulating IgE level HP:0003212, Multinodular goitre HP:0005987, Type II diabetes mellitus HP:0005978, Allergy HP:0012393, Eczema HP:0000964 |
| **70S^**^** | 60-70 | 70-80 | f | _ | | | Treatment refractory, disseminated mycobacterium avium infection. HSV2 and VZV infections. | | _ | _ | Unclassified immunodeficiencies | Autoantibodies against IFN-γ. CD4 T cell count↓ | Decreased proportion of CD4-positive T cells HP:0032218, Type II diabetes mellitus HP:0005978, Diarrhea HP:0002014, Lymphadenitis HP:0002840, Thyroid nodule HP:0025388 |
| **71S** | 0-10 | 20-30 | m | 1^st^ degree relative with unusual/severe or recurrent infection | | | Recurrent otitis media, pneumonia, and upper respiratory infections (bacterial) | | Bronchiectasis. Nephropathy. | VUS in *NLRP3* | Unclassified immunodeficiencies | Severely reduced B- cell count with normal levels of immunoglobulins. | Bronchiectasis HP:0002110, Nephropathy HP:0000112, B lymphocytopenia HP:0010976 |
| **72T** | 20-30 | 20-30 | f | _ | | | Molluscum contagiosum. Chronic vaginal candidiasis. | | Severe acne. Eczema. Melanosis coli. Skin abscess. Alopecia. | VUS in *UNC119,* VUS in *RIPK1* | Unclassified immunodeficiencies | IgG3↓. *Trueculture*: T-cell response↓ | Molluscum contagiosum HP:0032163, Recurrent vulvovaginal candidiasis HP:0012204, Stomatitis HP:0010280, Acne HP:0001061, Cutaneous abscess HP:0031292, Alopecia HP:0001596, Gingivitis HP:0000230, Recurrent urinary tract infections HP:0000010 |
| **73S** | 30-40 | 50-60 | m | _ | | | Recurrent infections of the skin and pneumonia (bacterial). | | Lymphedema and intestinal lymphangiectasia. | VUS in *NOD2,* VUS in *FLG* | Unclassified immunodeficiency es | IgG↓. CD4+ T-cell concentration↓ Naive T-cells ↓ | Lymphedema HP:0001004, Asthma: HP:0002099, Decreased circulating antibody level HP:0004313, Intestinal lymphangiectasia HP:0002593, Hypoalbuminemia HP:0003073, Erysipelas HP:0001055, T lymphocytopenia HP:0005403 |
| **Patients not fulfilling ESID (2019) Criteria** | | | | | | |  | |  |  |  |  |  |
| **74T^††^** | 10-20 | 10-20 | f | _ | | | _ | | Lymphadenitis. Recurrent ulcers of the mouth. AI. | P in *ERAP1^‡^* | Behçet’s Syndrome | N/A | Autoimmunity HP:0002960, Lymphadenitis HP:0002840, Oral ulcer HP:0000155 |
| **75S** | 0-10 | 50-60 | m | _ | | | Recurrent infections of the skin and pneumonia (Hemophilus influenzae, Staphylococcus Aureus, Pseudomonas aeruginosa). | | Asthma. Eczema. Hypohydrotic ectodermal dysplasia. | P in *EDA^§^,* | Hypohidrotic ectodermal dysplasia | Normal | Recurrent pneumonia HP:0006532, Hypohidrotic ectodermal dysplasia HP:0007607, Asthma: HP:0002099, Eczema HP:0000964 |
| **76S** | 40-50 | 40-50 | f | Relatives with asthma and allergy | | | Recurrent pneumonia (bacterial). | | Bronchiectasis. Asthma. Allergy. | _ | _ | Normal | Allergy: HP:0012393, Bronchiectasis HP:0002110, Asthma: HP:0002099, Peripheral neuropathy HP:0009830 |
| **77S** | 30-40 | 40-50 | f | _ | | | Recurrent pneumonia (haemophilus influenzae). | | _ | LP in *TNFRSF13B* | _ | Normal | Recurrent pneumonia HP:0006532 |
| **78S** | 30-40 | 30-40 | f | 1^st^ degree relatives with AI | | | Recurrent pneumonia and HPV infection. | | Autoimmunity. Abdominal pain. Episcleritis. | VUS in *NLRP1* | _ | Low B cells due to anti-CD20-antibody | Rheumatoid arthritis HP:0001370, Episcleritis HP:0100534, Decreased circulating antibody level HP:0004313, Recurrent pneumonia HP:0006532, Persistent human papillomavirus infection HP:0020114, Cataract HP:0000518 |
| **79S** | unknown | 50-60 | f | 1^st^ degree relative with unusual/severe or recurrent infection | | | Recurrent upper respiratory tract infections, pneumonia and sinusitis (bacterial). Recurrent HSV infections. | | AI. Recurrent abdominal pain. Angioedema. Allergy. Abscess. Focal epilepsy. Migraine. | _ | _ | Normal | Allergy: HP:0012393, recurrent infections: HP:0002719, autoimmunity: HP:0002960. Vitiligo HP:0001045. Focal motor seizure HP:0011153. Migraine HP:0002076 Psoriasiform dermatitis HP:0003765, Encephalitis HP:0002383 Duodenitis HP:0033117, Cholecystitis HP:0001082, Angioedema HP:0100665, Episodic abdominal pain HP:0002574 |
| **80S** | 20-30 | 20-30 | m | _ | | | Rhino orbital and pulmonary Mucormycosis. | | Acute hepatic failure. | VUS in *JAK3* | _ | Normal | Acute hepatic failure HP:0006554 |
| **81S** | 0-10 | 40-50 | f | 1^st^ degree relative with similar symptoms | | | Recurrent pneumonia, meningitis and sinusitis (bacterial). Recurrent abscess formation and skin infections. Diverticulosis with abdominal abscess formation. | | _ | VUS in *IL17F,* P in *BLK,* P in *BRIP1^§^* | _ | Normal | Recurrent bacterial infections HP:0002718, Recurrent abscess formation HP:0002722,  Psoriasiform dermatitis: HP:0003765. Bowel diverticulosis HP:0005222 |
| **82T** | 20-30 | 20-30 | f | 1^st^ degree relative with asthma | | | Recurrent upper respiratory tract infections. | | Recurrent chronic cough and dyspnoea. | _ | _ | Normal | Dyspnoea HP:0002094, Bicuspid aortic valve HP:0001647 |
| **83T** | 0-10 | 30-40 | f | _ | | | Recurrent otitis media and tonsillitis in childhood (bacterial) | | Severe asthma and liver disease in childhood (UNS). Behçet´s Disease. Bronchiectasis. Pulmonary insufficiency. | _ | _ | Normal | Pulmonary insufficiency HP:0010444, Asthma HP:0002099, Bronchiectasis HP:0002110, Osteopenia HP:0000938, Macular degeneration HP:0000608 |
| **84S** | 0-10 | 10-20 | m | _ | | | Recurrent tonsillitis | | Recurrent fever with swollen lymph nodes and aphthous stomatitis. Allergy. | VUS in *XIAP* | _ | N/A | Recurrent aphthous stomatitis HP:0011107, Recurrent fever HP:0001954, Allergy: HP:0012393. |
| **85S** | 30-40 | 30-40 | f | _ | | | _ | | Fatigue. Short bowel syndrome. Epigastric pain. Fever. Unintended weight loss. | _ | _ | N/A | Recurrent fever HP:0001954, Epigastric pain HP:0410019, Secondary Short Bowel Syndrome ORPHA:95427, Fatigue HP:0012378 |

^*^S: Singleton, T: Trio, F: Family, P: parent, C: child, A: Adult in family without included children. ^†^ACMG classification: 3 = variant of uncertain significance (VUS), 4 = likely pathogenic (LP), and 5 = pathogenic (P). ^‡^Variant causal for PID. ^§^Incidental finding(s). ^¶^Family is planned to be included in a prospective study focused on CVID. ^#^The case is published (PMID: 34037797). ^**^The case is published (PMID: 34692415). ^††^The case is published (PMID: 31873220).

Abbreviations: AI: Autoimmunity, HPO: Human phenotype ontology.

**Supplementary Table 3: Results of initial genetic analysis**

| **ID^*^** | **WES / WGS** | **Genetic result of SNV or SV analysis (gene, cDNA, Protein, RefSeq)^2, 3^** | **ACMG classification^†^** | | **SNV/SV predicted coding effect** | | **Zygosity** | **Inheritance** | **Allele frequency for NFE in GnomAD (%), and for SV DGV (%), (All)** | **CADD** | **Previous report of variant in HGMD (PMID)** | **Comment** |
| --- | --- | --- | --- | --- | --- | --- | --- | --- | --- | --- | --- | --- |
| **Unclassified Antibody Deficiency** | | |  | |  | |  |  |  |  |  |  |
| **4T** | WES | *NCF2* (c.1081A>T, p.(Thr361Ser), NM_000433.3) | 3 | | Missense | | Het | AR | 0.23 | 24.5 | PMID:  2393022 | The parents are each heterozygous for the *NCF2* variants. |
| **4T** | WES | *NCF2* (c.113G>A, p.(Arg38Gln), NM_000433.3) | 3 | | Missense | | Het | AR | 0.2 | 27.4 | PMID:  24931457 | The parents are each heterozygous for the *NCF2* variants. |
| **5S** | WES | *IL17RA* (c. 1A>C, p.(Met1?), NM_014339.6) | 4 | | Start loss | | Het | AR | 0 | 16.5 | _ | _ |
| **5S** | WES | *IL17RA* (c.152C>T, p.(Thr51Met), NM_014339.6) | 3 | | Missense | | Het | AR | 0.27 | 11.9 | _ | _ |
| **5S** | WES | *IL17RA* (c.958T>C, p.(Trp320Arg), NM_014339.6) | 3 | | Missense | | Het | AR | 0.29 | 24.9 | _ | _ |
| **9S** | WGS | *TNFRSF13B* (c.542C>A, p.(Ala181Glu), NM_012452.2) | 3 | | Missense | | Het | AR/AD | 0.94 | 16 | PMID:  16007087 | _ |
| **11S** | WES | *RNF168* (c.493C>T, p.(Arg165*), NM_152617.3)^‡^ | 4 | | Nonsense | | Ho | AR | 0.004 | 36 | _ | _ |
| **14F** | WGS | *NLRP2* (c.986T>G, p.(Ile329Ser), NM_001174083.2) | 3 | | Missense | | Het | AD | 0.18 | 17.5 | _ | The mother and 3 children are all heterozygous for the variant. |
| **15F** | WGS | *NLRP3* (c.2182A>G, p.(Ser728Gly), NM_004895.4) | 3 | | Missense | | Het | AD | 0.07 | 15.8 | PMID: 28421071 | The mother and 2 children are all heterozygous for the variant. |
| **Selective IgM Deficiency** | | |  | |  | |  |  |  |  |  |  |
| **16S** | WES | *TNFRSF13C* (c.475C>T, p.(His159Tyr), NM_052945.3) | 3 | | Missense | | Het | AR | 0.68 | 26.3 | PMID: 21041452 | _ |
| **IgG-Subclass Deficiency** | | |  | |  | |  |  |  |  |  |  |
| **17S** | WGS | *DOCK8* (c.1683C>A, p.(Asn561Lys), NM_203447.3) | 3 | | Missense | | Het | AR | 0.02 | 22.7 | _ | _ |
| **22S** | WGS | *CARD11* (c.3019+9C>T, NM_032415.5) | 3 | | Splice effect | | Het | AR/AD | 0.24 | _ | _ | _ |
| **23S** | WES | *IKZF1* (c.547C>T, p.(Arg183Cys), NM_006060.5) | 3 | | Missense | | Het | AD | 0 | 32 | _ | _ |
| **23S** | WES | *BRCA2* (c.5754_5755del, p.(His1918Glnfs*5), NM_000059.3)^§^ | 5 | | Frameshift | | Het | AR/AD | 0.001 | 26.5 | PMID:  21318380 | _ |
| **24S** | WGS | *MEFV* (c.2084A>G, p.(Lys695Arg), NM_000243.2) | 3 | | Missense | | Het | AR/AD | 0.93 | 14.4 | PMID: 31088470 | _ |
| **24S** | WGS | *IGLL1* (c.428C>T, p.(Pro143Leu), NM_001369906.1) | 3 | | Missense | | Het | AR | 0.06 | 15.0 | PMID: 33178177 | _ |
| **Complement Component 2 Deficiency** | | |  | |  | |  |  |  |  |  |  |
| **25S** | WGS | *C2* (c.841_849+19del, NM_000063.5)^‡^ | 4 | | Exon skipping, premature stop | | Ho | AR | 0.69 | 25.7 | PMID: 1577763 |  |
| **T-cell Deficiency and Combined Immunodeficiency** | | | | | |  |  |  |  |  |  |  |
| **29S** | WES | *STAT3* (c.307C>T, p.(Arg103Trp), NM_139276.3) | 3 | | Missense | | Het | AD | 0 | 29.5 | PMID: 27379089 | _ |
| **Pathogen-Specific Immunodeficiency** | | |  | |  | |  |  |  |  |  |  |
| **36S** | WGS | *TLR9* (c.853C>T, p.(Arg285Cys), NM_017442.3) | 3 | | Missense | | Het | Un-known | 0.005 | 23.4 | _ | _ |
| **36S** | WGS | *IL17F* (c.370C>T, p.(Gln124*), NM_052872.3) | 3 | | Nonsense | | Het | AD | 0 | 37 | _ | _ |
| **37S** | WGS | *C2* (c.841_849+19del, NM_000063.5) | 4 | | Exon skipping, premature stop | | Het | AR | 0.69 | 25.7 | PMID:  1577763 | _ |
| **39F** | WGS | *TLR3* (c.2553C>G, p.(Phe851Leu), NM_003265.2) | 3 | | Missense | | Het | AR/AD | ≤ 0.001 | 23 | _ | 1^st^ degree relative with resembling symptoms is heterozygous for the variant. |
| **40S** | WGS | *TINF2* (c.793C>T, p.(Arg265*), NM_001099274.1) | 3 | | Nonsense | | Het | AD | 0.006 | 35 | PMID: 30891747 | _ |
| **Familial Chronic Mucocutanous Candidiasis** | | |  | |  | |  |  |  |  |  |  |
| **44T** | WES | *STAT1* (c.1154C>T, p.(Thr385Met), NM_007315.4)^‡^ | 5 | | Missense | | Het | AD | 0 | 26.5 | PMID: 22730530 | *De Novo*. Patient has died. |
| **45S** | WES | *STAT1* (c.1204_1205delinsTT, p.Ala402Phe, NM_007315.3)^‡^ | 5 | | Missense | | Het | AD | 0 | 29.5 | PMID: 16868551 | Variant segregates with the family in several family members. |
| **46S** | WES | *STAT1* (c.823C>A, p.(Gln275Lys), NM_007315.3)^‡^ | 4 | | Missense | | Het | AD | 0 | 22.7 | _ | *De Novo.* |
| **Autoimmune Lymphoproliferative Syndromes** | | | |  |  | |  |  |  |  |  |  |
| **47T** | WES | *RAG1* (c.478C>T, p.(Arg160Trp), NM_000448.2) | 3 | | Missense | | Het | AR | ≤ 0.001 | 23.9 | PMID: 28216420 | *De Novo*. Patient has died. |
| **48T** | WES | *CTLA4* (c.410C>T, p.(Pro137Leu), NM_005214.5)^‡^ | 5 | | Missense | | Het | AD | 0 | 24.1 | PMID: 30377434 | *De Novo.* |
| **Autoinflammatory Disorders and Periodic Fever Syndromes** | | | | | |  |  |  |  |  |  |  |
| **52S** | WGS | *NLRP3* (c.598G>A, p.(Val200Met), NM_001079821.2) | 3 | | Missense | | Het | AD | 0.83 | <10 | PMID: 28137891 | Two 1^st^ degree relatives with resembling symptoms are both heterozygous for the variant. This variant was detected before WGS was performed in another laboratory. WGS confirmed the finding. |
| **53S** | WGS | *TCIRG1* (c.1297C>T. p.(Gln433*), NM_006019.3) | 5 | | Nonsense | | Het | AR | 0.0051 | 36 | PMID: 15300850 | _ |
| **53S** | WGS | *MEFV* (c.2084A>G, p.(Lys695Arg), NM_000243.2) | 3 | | Missense | | Het | AR/AD | 0.59 | 14.4 | PMID: 29080837 | _ |
| **53S** | WGS | *PLCG2* (c.1565C>G, p.(Pro522Arg), NM_002661.4) | 3 | | Missense | | Het | AD | 0.51 | 17.4 | PMID: 30568185 | _ |
| **54S** | WGS | *FLG* (c.2282_2285del, p.(Ser761Cysfs*36), NM_002016) | 5 | | Frameshift | | Het | AR/AD | 2.05 | 19.43 | PMID: 29068602 | _ |
| **61T** | WGS | *PLCG2* (c.1358T>C, p.(Ile453Thr), NM_002661.4) | 3 | | Missense | | Het | AR/AD | 0 | 26.5 | _ | The father is heterozygous for the variant. |
| **Defects of NFκ-B Signaling** | | |  | |  | |  |  |  |  |  |  |
| **62F** | WGS | *CASP8* (c.262del, p.(Arg88Glyfs*25), NM_033355.3) | 4 | | Frameshift | | Het | AR | 0 | <10 | _ | The father of 62FP, 62FP and 62FC are each heterozygous for the variant. The case is published  (PMID: 34037797). |
| **62F** | WGS | *IKBKB* (c.1227T>G, p.(Ser409Arg), NM_001556.2) | 3 | | Missense | | Het | AR/AD | 0 | 17.6 | _ | The father of 62FP, 62FP and 62FC are each heterozygous for the variant. |
| **62F** | WGS | *IRAK1* (c.137-19G>A, NM_001569.4) | 3 | | Splice | | Chet | XLR | 0.63 | 15.1 | _ | The father of 62FP and 62FC are each hemizygous for the variant. 62FP is heterozygous for the variant. |
| **62F** | WGS | *IRAK1* (c.1637C>T, p.(Thr546Met), NM_001025243.2) | 3 | | Missense | | Chet | XLR | 0.66 | 13.9 | _ | The mother of 62FP and 62FP are each heterozygous for the variant. 62FC does not carry the variant. |
| **63S** | WGS | *NFKB1* (exons 9-12) (c.730+439_1211-602del, p.(Lys244_Gly404delinsArg), NM_003998.3)^‡^ | 4 | | In-frame deletion | | Het | AD | 0 | _ | _ | ~14,8 Kb |
| **Thymoma with Immunodeficiency** | | |  | |  | |  |  |  |  |  |  |
| **64S** | WES | *NFKB2* (c.472C>T, p.(Arg158Trp), NM_002502.5) | 3 | | Missense | | Het | AD | 0.002 | 23 | _ | _ |
| **Unclassified Immunodeficiency** | | |  | |  | |  |  |  |  |  |  |
| **71S** | WGS | *NLRP3* (c.380T>C, p.(Ile127Thr), NM_001079821.3) | 3 | | Missense | | Het | AD | 0.001 | <10 | _ | _ |
| **72T** | WGS | *UNC119* (c.487G>T, p.(Glu163*), NM_005148.3) | 3 | | Nonsense | | Het | AD | 0.001 | 38 | _ | The mother is heterozygous for the variant. |
| **72T** | WGS | *RIPK1* (c.521A>C, p.(Asn174Thr), NM_001354930.2) | 3 | | Missense | | Het | AD/AR | 0.005 | 17.8 | _ | The father is heterozygous for the variant. |
| **73S** | WES | *NOD2* (c.1292C>T, p. (Ser431Leu), NM_022162.2) | 3 | | Missense | | Het | AD | 0.14 | 17.2 | PMID: 28422189 | _ |
| **73S** | WES | *NOD2* (c.2377G>A, p.(Val793Met), NM_022162.2) | 3 | | Missense | | Het | AD | 0.17 | 21.9 | PMID: 28422189 | _ |
| **73S** | WES | *FLG* (c.2929C>T, p.(Gln977*), NM_002016.1) | 3 | | Nonsense | | Het | AD/AR | 0.02 | 40 | PMID: 16444271 | _ |
| **Patients not fulfilling ESID (2019) Criteria** | | |  | |  | |  |  |  |  |  |  |
| **74T** | WES | *ERAP1* (c.-137del, RS140416843)^‡^ | 5 | | Regulator effect | | Ho | AR | 0.025 | _ | PMID:  23291587 | The parents are each heterozygous for the variant. |
| **77S** | WGS | *TNFRSF13B* (c.542C>A, p.(Ala181Glu), NM_012452.2) | 4 | | Missense | | Het | AR/AD | 0.94 | 16 | PMID: 27379089 | _ |
| **78S** | WGS | *NLRP1* (c.2693G>A, p.(Arg898Gln), NM_033004.3) | 3 | | Missense | | Het | AD | 0.019 | 15.3 | _ | _ |
| **80S** | WGS | *JAK3* (c.2444C>T, p.(Thr815Met), NM_000215.3) | 3 | | Missense | | Het | AR | 0.002 | 24.3 | _ | _ |
| **81S** | WGS | *IL17F* (c.3G>C, p.(Met1?), NM_052872.3) | 3 | | Start loss | | Het | AD | 0.004 | 11.5 | _ | _ |
| **81S** | WGS | *BLK* (c.892C>T, p.(Arg298*), NM_001715.2) | 5 | | Nonsense | | Het | AD | 0.007 | 41 | _ | _ |
| **81S** | WGS | *BRIP1* (c.2400C>G, p.(Tyr800*), NM_032043.2)^§^ | 5 | | Nonsense | | Het | AR/AD | 0.004 | 33 | PMID: 30322717 | _ |
| **84S** | WGS | *XIAP* (c.1408A>T, p.(Thr470Ser), NM_001167.3) | 3 | | Missense | | Hem | XLR | 0.07 | 21.2 | PMID: 21119115 | _ |

^*^S: Singleton, T: Trio, F: Family, P: Parent, C: Child, A: Adult in family without included children. ^†^ACMG classification: 3 = variant of uncertain significance (VUS), 4 = likely pathogenic (LP), and 5 = pathogenic (P). ^‡^Variant causal for PID. ^§^Incidental finding(s).

Abbreviations: AD: Autosomal dominant. AR: Autosomal recessive. CADD: Combined Annotation Dependent Depletion. Chet: Compound Heterozygous. Het: Heterozygous. Hem: Hemizygous. HGMD: Human Gene Mutation Database. Ho: Homozygous. NFE: GnomAD Non-Finish European population (All patients with genetic findings are NFE). PMID: PubMed Identifier. SNV: Single nucleotide variant or small indel, SV: Structural variant, WES: Whole Exome Sequencing. WGS: Whole Genome Sequencing. XLR: X-linked Recessive.
